# Differential reactivity of SARS‐CoV‐2 S‐protein T‐cell epitopes in vaccinated versus naturally infected individuals

**DOI:** 10.1002/cti2.70031

**Published:** 2025-05-06

**Authors:** Daniel J Browne, Pauline Crooks, Corey Smith, Denise L Doolan

**Affiliations:** ^1^ Centre for Molecular Therapeutics, Australian Institute of Tropical Health and Medicine James Cook University Cairns QLD Australia; ^2^ QIMR Berghofer Centre for Immunotherapy and Vaccine Development and Translational and Human Immunology Laboratory, Department of Immunology QIMR Berghofer Medical Research Institute Brisbane QLD Australia; ^3^ Faculty of Medicine The University of Queensland Brisbane QLD Australia; ^4^ Institute for Molecular Bioscience The University of Queensland St Lucia QLD Australia

**Keywords:** COVID‐19 vaccines, HLA‐B7 antigen, SARS‐CoV‐2, spike glycoprotein, T‐cell epitopes, T‐lymphocyte

## Abstract

**Objectives:**

Vaccine‐induced protective immunity against SARS‐CoV‐2 has proved difficult to sustain. Robust T‐cell responses are thought to play an important role, but T‐cell responses against the SARS‐CoV‐2 spike protein (S‐protein), the core vaccine antigen, following vaccination or natural infection are incompletely understood.

**Methods:**

Herein, the reactivity of 170 putative SARS‐CoV‐2 S‐protein CD8^+^ and CD4^+^ T‐cell peptide epitopes in the same individuals prior to vaccination, after COVID‐19 vaccination, and again following subsequent natural infection was assayed using a high‐throughput reverse transcription‐quantitative PCR (HTS‐RT‐qPCR) assay.

**Results:**

The profile of immunoreactive SARS‐CoV‐2 S‐protein epitopes differed between vaccination and natural infection. Vaccine‐induced immunoreactive epitopes were localised primarily into two extra‐domanial regions. In contrast, epitopes recognised following natural infection were spread across the antigen. Furthermore, T‐cell epitopes in naïve individuals were primarily recognised in association with HLA‐A, while natural infection shifted epitope associations towards HLA‐B, particularly the B7 supertype.

**Conclusion:**

This study provides insight into T‐cell responses against the SARS‐CoV‐2 S‐protein following vaccination and subsequent natural infection.

## Graphical abstract

We investigated the immunoreactivity of SARS‐CoV‐2 spike protein T‐cell epitopes in individuals who were SARS‐CoV‐2 naive, vaccinated with the AstraZeneca vaccine, and those who became naturally infected after vaccination. While the number of immunoreactive epitopes remained consistent across all groups, we observed a shift in T‐cell responses: naive individuals predominantly recognised HLA‐A‐associated epitopes, vaccination clustered immunoreactive epitopes within two specific regions of the spike protein, and natural infection shifted immunoreactivity towards HLA‐B‐associated epitopes, particularly the B7 supertype. These findings suggest that vaccination and natural infection elicit different T‐cell responses, providing insights that could inform future vaccine design to enhance long‐term immunity against COVID‐19.

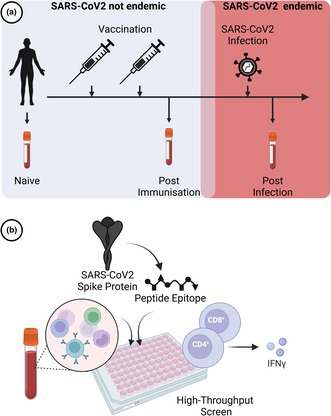

## Introduction

The SARS‐CoV‐2 coronavirus, the causative agent of COVID‐19,^1^ is now endemic throughout the world, despite the rapid development of effective vaccines that induce robust immunity against the virus.^2^ While vaccination against SARS‐CoV‐2 has substantially reduced the mortality and morbidity associated with COVID‐19,^3^, ^4^, ^5^ the effectiveness of the available vaccines decreases relatively rapidly,^6^, ^7^ and multiple novel viral variants have emerged that can evade vaccine‐induced protection.^8^ By 2023, individuals who received the original wild‐type COVID‐19 vaccines were found to have minimal protection against severe disease requiring hospital admission.^9^ This waning immunity contrasts with the sustained protection provided by many vaccines included in global adolescent immunisation schedules, such as measles, mumps, rubella and hepatitis B.^10^, ^11^ Following convalescence from exposure to SARS‐CoV‐2, natural immunity provides robust protection against reinfection and COVID‐19‐related hospitalisation, with this protection remaining relatively high over a longer period than vaccination.^12^, ^13^, ^14^, ^15^ Indeed, hybrid immunity, resulting from vaccination followed by subsequent natural infection, appears to provide the highest level of protection.^15^

Many COVID‐19 vaccines, including the Pfizer‐BioNTech mRNA vaccine (BNT162b2) and the Moderna mRNA vaccine (mRNA‐1273), were intended to induce high levels of neutralising antibodies.^16^ Conversely, the AstraZeneca vaccine (ChAdOx1 nCoV‐19) was designed to induce a balanced immune response that includes activated T‐cell responses in addition to antibody responses.^17^, ^18^ Both vaccine strategies proved effective at inducing robust antibody and T‐cell responses, but vaccine‐induced efficacy decreased relatively quickly for both.^19^ Promoting robust cellular immunity and cellular immune memory, particularly T‐cell‐mediated immunity, is expected to enhance the development of long‐term protection and maintain protection against novel viral variants.^20^, ^21^, ^22^ Broadly, CD8^+^ T cells can eliminate virally infected host cells,^23^ while T helper CD4^+^ T cells play a multipurpose role, including assisting in the production of high‐affinity neutralising antibodies or promoting immune activation, regulation and memory formation.^24^ However, the specificity, magnitude and kinetics of T‐cell reactivity to SARS‐CoV‐2 in relation to vaccination or natural infection are incompletely understood.

The magnitude of T‐cell responses has been associated with the effectiveness of host immunity to SARS‐CoV‐2 infection.^25^ Perturbations to T‐cell populations and circulating numbers^26^ and a decline in naïve T‐cell number and diversity^27^ have been all associated with poor COVID‐19 clinical outcomes. Several HLA alleles have been identified as beneficial for immunity to SARS‐CoV‐2,^15^, ^28^ while others have been found to be detrimental^29^, ^30^, ^31^ to patient outcomes. Immunodominance is the ‘choice of the immune system’ to develop immunity to any specific antigen or epitope,^32^ and only a fraction of potential peptide epitopes induce measurable cellular immunity.^33^ The tripartite interaction of expressed HLA alleles, antigen and peptide epitope chemistry, and the repertoire of available naïve T cells is the dominant paradigm that is believed to determine epitope immunodominance.^34^ Understanding T‐cell immunodominance during a SARS‐CoV‐2 infection following vaccination and subsequent natural infection may provide insight to develop more efficacious vaccines.

Currently, all licensed COVID‐19 vaccines are based on the SARS‐CoV‐2 spike protein (S‐protein) antigen. The S‐protein is a large homotrimer transmembrane glycoprotein that facilitates SARS‐CoV‐2 entry by binding to the human AE2 receptor. Each trimer consists of between 1273 and 1300 amino acids, depending on the viral variant,^35^ which allows for a large number of potential T‐cell epitopes and abundant T‐cell peptide epitope–HLA presentation.^36^ Various studies have explored S‐protein epitope immunodominance or reactivity following vaccination,^37^, ^38^, ^39^ and in infected or convalescent patients.^31^, ^40^, ^41^, ^42^, ^43^, ^44^, ^45^ Pre‐existing T‐cell immunity to SARS‐CoV‐2 in COVID‐19 naive individuals is another notable phenomenon observed during the COVID‐19 pandemic,^41^, ^46^ pre‐existing presumably from exposure to other endemic coronaviruses.^47^ Further developing an understanding of which SARS‐CoV‐2 S‐protein T‐cell epitopes are immunodominant in pre‐existing immunity, following vaccination and following an infection may provide critical insight for future vaccine design.

The identification of immunodominant T‐cell epitopes within the SARS‐CoV‐2 S‐protein involves analysis of peptide‐stimulation reactivity by human peripheral blood mononuclear cells (PBMCs), quantified by measuring markers of activation, such as the secretion of interferon gamma (IFN‐γ).^48^ T‐cell epitopes can be identified by screening large panels of overlapping peptides representing the complete protein or a more focused panel of putative peptide epitopes predicted using software, such as the T‐cell epitope NetMHCpan HLA‐peptide‐binding prediction tool^49^ within the Immune Epitope Database (IEDB).^50^ In this study, 170 CD8^+^ and CD4^+^ T‐cell peptide epitopes were identified from the SARS‐CoV‐2 S‐protein, based on prediction to bind with high affinity to a range of class I and class II HLA alleles using the IEDB NetMHCpan algorithm. Thousands of peptides were identified overall and were subsequently prioritised by reported immunogenicity^21^, ^41^, ^43^, ^46^, ^51^, ^52^ (as well as HLA‐peptide binding affinity) to define a subset of putative T‐cell epitopes for study.

Peptide epitopes are typically screened for immunoreactivity with conventional assays, including IFN‐γ ELISpot, intracellular cytokine staining (ICS) or activation‐induced marker (AIM) assay.^53^, ^54^, ^55^ These assays typically require a high number of PBMCs, especially when screening the large number of potential peptide epitopes available within the SARS‐CoV‐2 S‐protein. To address this limitation, we developed a sensitive and specific high‐throughput screening reverse transcription‐quantitative PCR (HTS‐RT‐qPCR) assay to screen large panels of putative T‐cell epitope peptides from low numbers of PBMCs.^56^ Herein, we applied this assay to identify immunoreactive T‐cell epitopes from SARS‐CoV‐2 recognised by humans naïve to SARS‐CoV‐2 (pre‐existing immunity, cross‐reactive to other viruses), following COVID vaccination and following SARS‐CoV‐2 infection. We used our HTS‐RT‐qPCR assay to evaluate the immunoreactivity of these peptide epitopes in donors either naïve to the S‐protein, vaccinated with the S‐protein or following a subsequent natural infection. Where available, matched PBMCs collected from donors' pre‐exposure, post‐vaccination and post‐infection were tested. This study contributes to the understanding of vaccine epitope immunodominance and kinetics, while highlighting the utility of HTS‐RT‐qPCR for peptide epitope immunoreactivity testing.

## Results

### All donors developed S‐protein‐specific antibodies but, as expected, none produced nucleocapsid‐specific antibodies after receiving the S‐protein‐based SARS‐CoV‐2 vaccine

Following vaccination and subsequent natural infection, SARS‐CoV‐2 S‐protein antibody titres increased in all donors, except Donor 3 (Supplementary figure 1). Antibody titres specific to the NCAP remained low after vaccination and rose only upon infection in all donors except for Donor 3, whose NCAP titres stayed low (Supplementary figure 1). These findings indicate that double homologous AstraZeneca vaccination induced robust S‐protein antibody responses, and that NCAP‐specific antibodies only appear after infection with SARS‐CoV‐2, as expected. Donor 3, despite self‐reporting COVID‐19, may not have been infected or failed to seroconvert.

### The number of immunoreactive T‐cell epitopes was generally consistent across donor immune status

We defined a list of 170 putative SARS‐CoV‐2 S‐protein peptide epitopes (Supplementary table 1) from a total of 7500 identified CD8^+^ T‐cell peptide epitope–HLA allele combinations (Supplementary figure 2); test peptides were generally restricted to either the HLA‐A2, HLA‐A3/11, HLA‐A24, HLA‐B7 or HLA‐B8 MHC Class I (Supplementary figure 3) or Class II HLA‐DR or HLA‐DQ supertypes. These peptide epitopes were derived from the Wuhan reference strain, and analysis of sequence similarity for other SARS‐CoV‐2 variants was assessed and showed that the peptide epitope sequences were mostly homologous to clinically relevant circulating SARS‐CoV‐2 variants but not homologous to other circulating coronaviruses (Supplementary figure 4). Peptides were prioritised firstly by the sum of the response as identified from Tarke *et al*.,^43^ then by their IEDB predicted binding score (Supplementary table 1).

To study the magnitude and kinetics of T‐cell reactivity to these peptide epitopes in pre‐vaccinated, post‐vaccinated and post‐SARS‐CoV‐2 infected individuals, we used our published HTS‐RT‐qPCR assay^56^ to determine the expression of *IFN‐γ* mRNA from PBMCs stimulated with these peptides. Defining the threshold of positivity as a doubling of IFN‐γ expression (ΔΔ*C*_t_ > 2), we identified 65/170 epitopes that were immunoreactive in at least one donor. Robust T‐cell epitope reactivity was identified in SARS‐CoV‐2 S‐protein naïve individuals (17.0% positive: 29/170 epitopes, *n* = 9; Table 1), following vaccination (14.7% positive: 25/170 epitopes, *n* = 10; Table 1) and following subsequent natural exposure (16.5% positive: 28/170 epitopes, *n* = 8; Table 1), which was generally consistent with previously reported peptide immunogenicity amongst donors of similar immune status (Supplementary table 3).

**Table 1:** Categorical analysis of SARS‐CoV‐2 S‐protein epitope immunoreactivity and kinetics in matched donors following vaccination and natural infection

Immunoreactive epitopes	Immune status	Positive	Negative	Total	Positive (%)	*P*‐value *vs*. naive	*P*‐value *vs*. vaccination
*Peptide epitope immunoreactivity*							
Background	Naïve	29	141	170	17.0%	–	–
	Vaccinated	25	145	170	14.7%	0.6565	–
	Naturally infected	28	142	170	16.5%	> 0.9999	0.7652
						*P*‐value *vs*. immune status	*P*‐value *vs*. background
*Positive epitope kinetics*							
Naïve	Vaccinated	9	20	29	31.0%	–	0.0570
	Naturally infected	2	27	29	6.9%	0.0411	0.2634
Vaccinated	Naïve	9	16	25	36.0%	–	0.0330
	Naturally infected	6	19	25	24.0%	0.5380	0.3965
Naturally Infected	Naïve	2	26	28	6.9%	–	0.3997
	Vaccinated	6	22	28	24.0%	0.2516	0.2631

PBMCs (*n* = 12) were collected from donors who were either SARS‐CoV‐2 S‐protein naïve (Naïve), following COVID‐19 vaccination (Vaccinated) or following infection with SARS‐CoV‐2 (Naturally infected). The discovery rate of positive epitopes (% Positive) across all stimulations (Background) was determined from the relative number of reactive stimulations to non‐reactive stimulations. Positive epitope kinetics tested whether positive epitopes were consistently positive across classes of immune status by comparing the discovery rate of positive epitopes to background.

**Figure 1:**
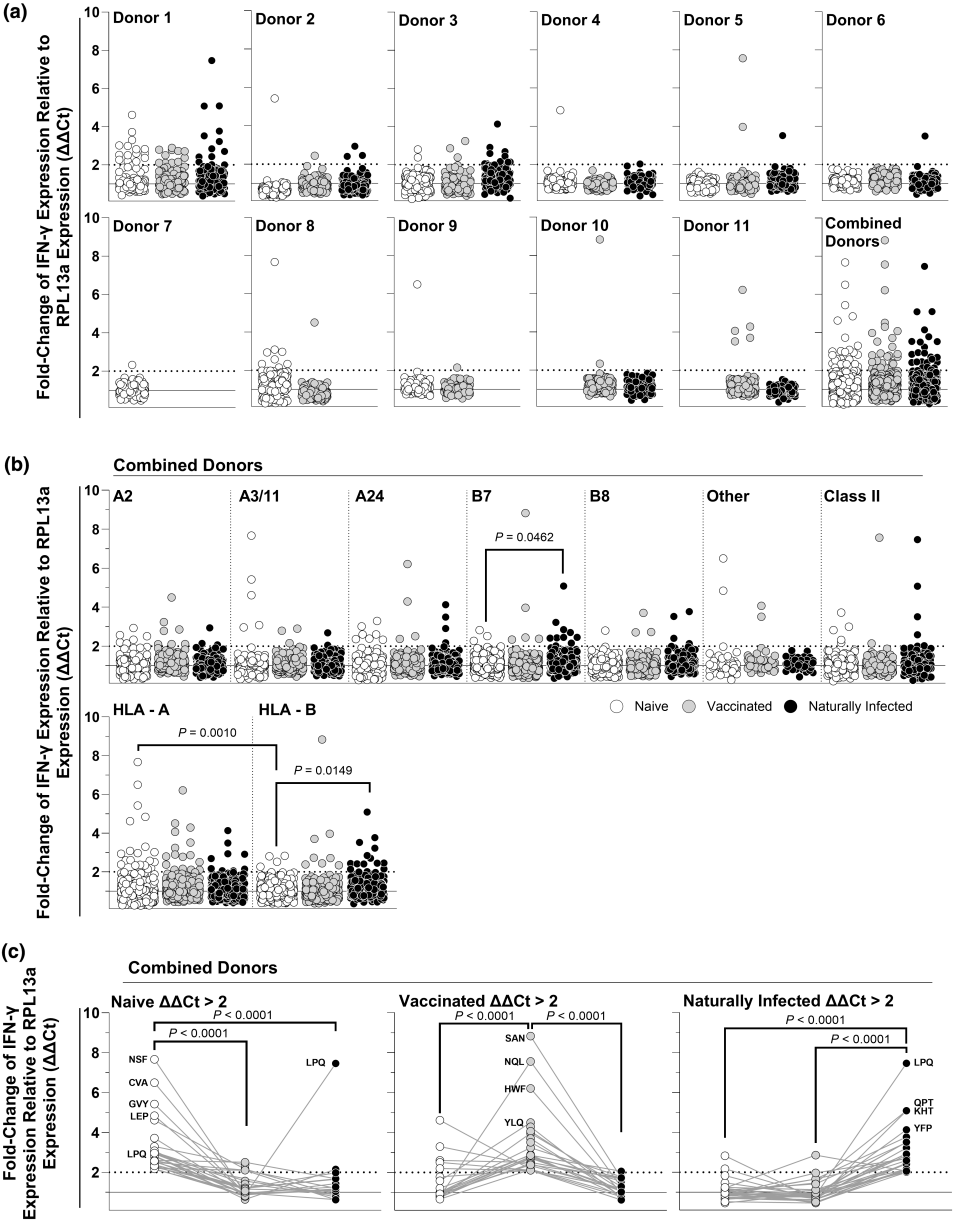
Peptide epitope immunoreactivity in peripheral blood mononuclear cells (PBMCs) isolated from individuals naïve to SARS‐CoV‐2, as well as following vaccination and natural infection. Interferon gamma (IFN‐γ) expression quantified through a high‐throughput RT‐qPCR (HTS‐RT‐qPCR) assay with fold change (ΔΔ*C*_t_) determined relative to the endogenous control reference gene Ribosomal Protein L13a (RPL13a) of PBMCs isolated from 12 individuals naïve to SARS‐CoV‐2 S‐protein (naïve; white dots), following homologous AstraZeneca COVID‐19 vaccination (vaccinated; grey dots), and following infection with SARS‐CoV‐2 (naturally infected; black dots) stimulated with 170 SARS‐CoV‐2 S‐protein peptide epitopes. Shown are data sorted by donor **(a)**, sorted into HLA‐A2, HLA‐A3/11, HLA‐A24, HLA‐B7, HLA‐B8, other and Class II supertype classifications **(b)**, and data showing matched peptide kinetics tracking immunoreactive epitopes (ΔΔ*C*_t_ > 2) across the naïve, vaccinated and naturally infected **(c)**. Epitopes with ΔΔ*C*_t_ between 0 and 10 shown graphically while all data were considered for statistical analysis. Selected immunoreactive peptides shown with three‐letter codes.

No significant differences were detected in the number of positive epitopes between naïve, vaccinated or naturally infected donors (peptide epitope immunoreactivity: *P* = 0.6565 (naïve *vs*. vaccinated), *P* > 0.9999 (naïve *vs*. naturally infected) and *P* = 0.7652 (vaccinated *vs*. naturally infected); Table 1). When considering individual donors, the number of reactive epitopes amongst donor‐matched PBMCs was generally consistent (Figure 1a), although some significant differences were detected. Specifically, when tested with a Fisher's exact test, there was a statistically significant increase in the number of reactive epitopes between naïve and naturally infected PBMCs (*P* = 0.0353) and a decrease in the number of reactive epitopes between naïve and vaccinated PBMCs (*P* = 0.0366) from Donors 3 and 8, respectively (Supplementary table 4). Taken together, these data demonstrate that the number of immunoreactive epitopes were generally consistent across donor immune status.

### High‐affinity HLA‐A epitopes are more immunoreactive in the naïve, while HLA‐B epitopes are more immunoreactive post‐natural infection

When considering the HLA supertype restriction element of the tested peptides (Figure 1b), there was no statistically significant increase in the number of reactive epitopes in HLA‐matched peptides as determined by Fisher's exact test (Supplementary table 5), except for an increase in immunoreactive HLA‐B7‐supertype epitopes between naïve and naturally infected PBMCs (*P* = 0.0462; Figure 1b). For epitopes predicted to bind with high affinity to any of the HLA‐A gene alleles, there were no statistically significant differences in the number of immunoreactive epitopes across donor immune status by Fisher's exact testing (Supplementary table 5). However, for epitopes predicted to bind with high affinity to HLA‐B gene alleles, there was a statistically significant increase in the number of immunoreactive epitopes between the naïve and the naturally infected (*P* = 0.0149; Figure 1b). Furthermore, when comparing across HLA genes, there was a statistically significant decrease in the number of immunoreactive epitopes in the naïve between HLA‐A‐ and HLA‐B‐associated epitopes (*P* = 0.0010; Figure 1b). These data demonstrate that the type of epitope recognised altered from HLA‐A restricted epitopes being predominantly immunoreactive in the naïve to HLA‐B restricted epitopes being predominantly immunoreactive following natural infection, with a particular focus on HLA‐B7 supertype epitopes.

### Peptide epitopes immunoreactive following vaccination were more likely to be immunoreactive in the naïve, but not following natural infection

We examined the kinetics of epitope immunoreactivity across donor‐matched naïve, vaccinated or naturally exposed samples (Figure 1c). There was a statistically significant decrease (*P* < 0.0001, in all cases) in immunogenicity for epitopes that were found to be immunoreactive in the naïve (naïve ΔΔ*C*_t_ > 2), vaccinated (vaccinated ΔΔ*C*_t_ > 2) or naturally infected (naturally infected ΔΔ*C*_t_ > 2) subjects, as determined by a non‐parametric Kruskal–Wallis test. Dunn's corrected multiple comparisons testing found there was a statistically significant decrease in immunogenicity at all matched kinetic timepoints (*P* < 0.0001 in all cases; Figure 1c). These data demonstrated that the immunoreactivity of epitopes varied between immune statuses.

To investigate whether there was an association between positive epitopes across immune status, we next investigated whether epitopes that were positive at one kinetic timepoint were also positive at another. Amongst the 29 epitopes identified as positive in the naïve, nine (31.0%) were also positive in the vaccinated, while two (6.9%) were positive in the naturally infected (positive epitope kinetics: Table 1). This difference was statistically significant by Fisher's exact testing (positive (%): 31.0% *vs*. 6.9%, *P* < 0.0411; Table 2), while neither were significantly different to the background rate of detection (positive (%): 17.0% *vs*. 31.0% *P* = 0.0570 (vaccinated); 14.7% *vs*. 6.9% *P* = 0.2634 (naturally infected)). Of the 25 epitopes that were immunoreactive in the vaccinated, nine (31.0%) were also immunoreactive in the naïve, while six (24.0%) were immunoreactive in the vaccinated. This difference was not statistically significant by Fisher's exact test (positive (%): 31.0% *vs*. 6.9%, *P* < 0.5380; Table 2), while there was a significant increase in the rate of positive epitopes between the background rate of detection and epitopes positive in the naïve and vaccinated (positive (%): 17.0% *vs*. 36.0% *P* = 0.0330; Table 2), but not in the vaccinated and naturally infected (positive (%): 16.5% *vs*. 24.0% *P* = 0.3965; Table 2). There was no statistically significant difference between the rates of detection of the six (24.0%) or two (6.9%) of 28 epitopes that were immunoreactive in the naturally infected and immunoreactive in the naïve or vaccinated, respectively (positive (%): 24.0% *vs*. 6.9%, *P* < 0.2516; Table 1), and no significant difference in these rates of detection when compared to background. There were no epitopes identified that were immunoreactive at all three timepoints. These data demonstrate there was a consistency of epitopes that were immunoreactive between matched donors that were SARS‐CoV‐2 spike protein naïve and who had been vaccinated.

**Table 2:** Categorical analysis of immunoreactive epitope localisation within SARS‐CoV‐2 S‐protein domains and regions following vaccination and natural infection

Domain	Immune status	Epitope in domain	Epitope outside domain	Total	In domain (%)	*P*‐value *vs*. naive	*P*‐value *vs*. vaccination
NTD	Naïve	13	17	30	43.3%	–	
	Vaccinated	4	22	26	15.4%	0.0401	–
	Naturally infected	10	19	29	34.5%	0.5959	0.1302
RBD	Naïve	5	25	30	16.7%	–	
	Vaccinated	3	23	26	11.5%	0.7116	–
	Naturally infected	3	26	29	10.3%	0.7065	> 0.9999
VC1	Naïve	4	26	30	13.3%	–	
	Vaccinated	7	19	26	26.9%	0.3130	–
	Naturally infected	4	25	29	13.8%	> 0.9999	0.3153
VC2	Naïve	5	25	30	16.7%	–	
	Vaccinated	11	15	26	42.3%	0.0426	–
	Naturally infected	4	25	29	13.8%	> 0.9999	0.0321
VC1/VC2 combined	Naïve	9	21	30	30.0%	–	
	Vaccinated	18	8	26	69.2%	0.0068	–
	Naturally infected	8	21	29	27.6%	> 0.9999	0.0029

The location of immunoreactive epitopes (ΔΔ*C*_t_ > 2) was tested relative to the number of epitopes inside and outside of domains across combined (*n* = 12) donors of various immune statuses. Tested were the N‐terminus domain (13–304aa; NTD), the Receptor Binding (319–541aa; RBD), Vaccination Cluster 1 (590–730aa; VC1), Vaccination Cluster 2 (905–1115aa; VC2) and epitopes within both VC1 and VC2 (Combined).

Given this consistency of epitopes between the naïve and vaccinated donors, we sought to investigate whether the epitopes immunoreactive in donors of various immune statuses were associated with SARS‐CoV‐2 variant homology. Of the epitopes selected for this study, 38 of 170 (22.3%) had less than 100% homology to all clinically circulating SARS‐CoV‐2 variants (Supplementary figure 4), while 8 of 29 (27.6%), 8 of 25 (32.0%) and 10 of 27 (37.0%) peptides that induced an immunogenic response in the naïve, vaccinated and naturally infected, respectively, did not have 100% homology (Supplementary table 6). There was no significant difference in the number of epitopes with imperfect homology by Fisher's exact testing, either when compared to the total peptides or across donor immune status. These data suggest that 100% epitope sequence homology did not play a significant role in determining epitope immunogenicity.

### The localisation of immunoreactive peptide epitopes within the SARS‐CoV‐2 S‐protein is dependent upon immune status

**Figure 2:**
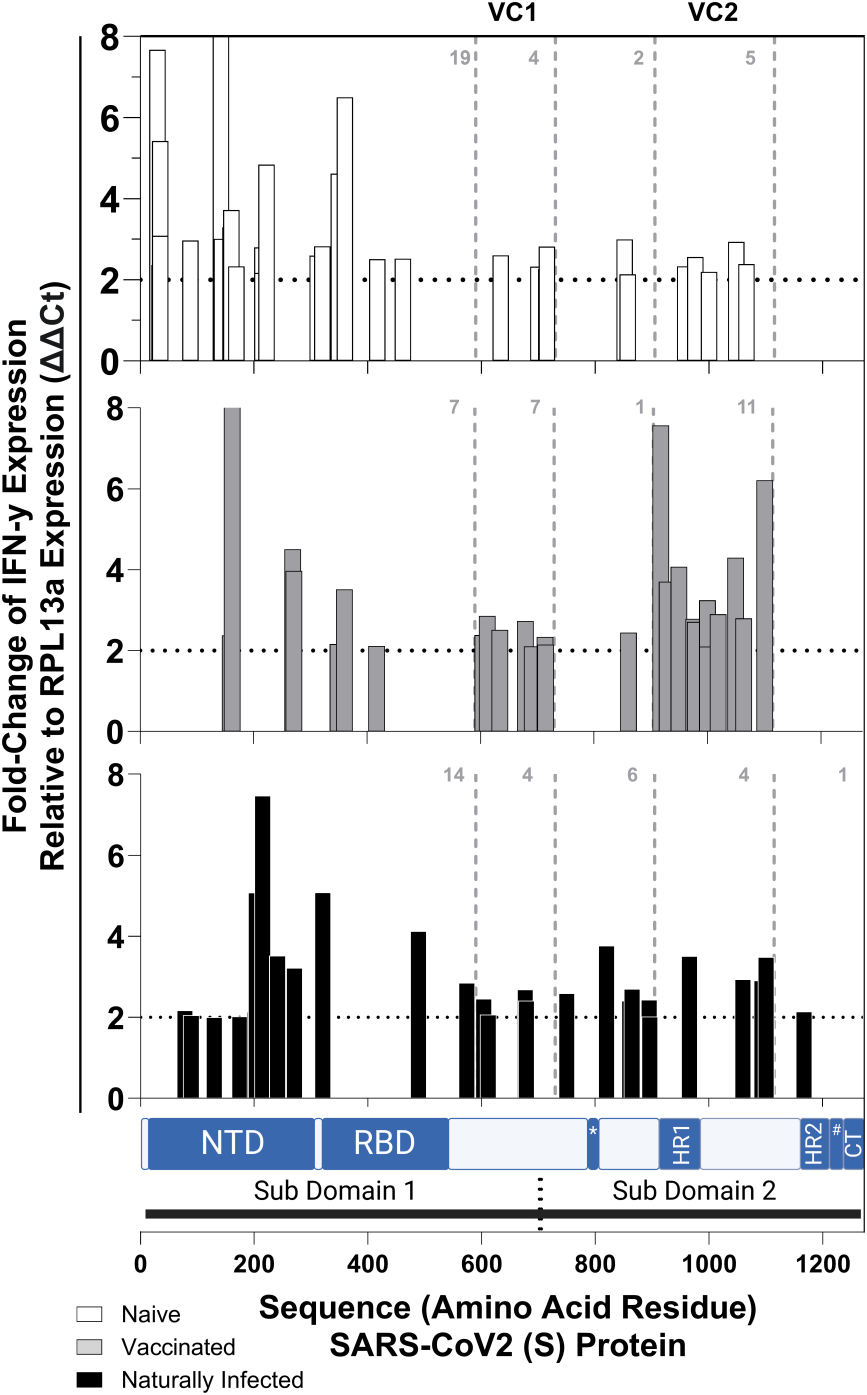
Immunoreactive peptide epitope locations along the SARS‐CoV‐2 spike (S) protein amino acid sequence. Immunoreactivity was determined as interferon gamma (IFN‐γ) expression quantified through a high‐throughput RT‐qPCR (HTS‐RT‐qPCR) assay with fold change (ΔΔ*C*_t_) relative to the endogenous control reference gene Ribosomal Protein L13a (RPL13a) of peripheral blood mononuclear cells (PBMCs) isolated from 12 individuals naïve to SARS‐CoV‐2 S‐protein (naïve; white bars), following homologous AstraZeneca COVID‐19 vaccination (vaccinated; grey bars) and following infection with SARS‐CoV‐2 (naturally infected; black bars) stimulated with 170 SARS‐CoV‐2 S‐protein peptide epitopes. Discrete locations shown along the amino acid sequence are Sub‐Domain 1 (1–681) and Sub‐Domain 2 (686–1273aa), and the N‐terminus (13–304aa; NTD), Receptor Binding (319–541aa; RBD), internal fusion peptide (816–833; *), Heptad Repeat 1 (981–983aa; HR1), Heptad Repeat 2 (1162–1203aa; HR2), transmembrane (1213–1237aa; #); Cytoplasmic Tail (1274 to the end of the protein; CT) domains. Vaccination Cluster 1 (590–730aa; VC1) and Vaccination Cluster 2 (905–1115aa; VC2) were experimentally defined by a high density of immunoreactive epitopes in these regions in donors following vaccination (grey bars). Numbers shown are total immunoreactive (ΔΔ*C*_t_ > 2) epitopes identified within VC1 and VC2 defined regions.

We next sought to determine whether there was variation in the location of immunoreactive epitopes along the SARS‐CoV‐2 S‐protein amino acid sequence amongst donors of either naïve, vaccinated or naturally exposed immune status. Broadly, epitopes appeared to cluster, especially in the vaccinated (Figure 2). This contrasted with the spread of the 170 selected peptide epitopes, which were generally spread across the S‐protein sequence (Supplementary figure 2). Fisher's exact testing found there was a statistically significant decrease in the number of immunoreactive epitopes in the N‐terminus domain (NTD) following vaccination. Specifically, of the 30 stimulations that were immunoreactive in the naïve, 13 (43.3%) were from peptide epitopes found in the NTD. While of the 26 stimulations identified as immunoreactive following vaccination, only four (15.4%) epitopes were in the NTD (in domain (%): 43.3% *vs*. 15.4%, *P* = 0.0401; Table 2). There was no significant difference in the number of immunoreactive epitopes in the NTD between the naturally infected and naïve (in domain (%): 34.5% *vs*. 43.3%; *P* = 0.5959; Table 2) or the naturally infected and vaccinated (in domain (%): 34.5% *vs*. 15.4%; *P* = 0.1302; Table 2). Furthermore, there were no significant differences in epitope location amongst donors of differing immune status within the receptor‐binding domain (Table 2), or any other previously defined tested domains (Supplementary table 7). These data demonstrate that while epitope localisation differed in the vaccinated, it was generally not associated with previously defined domains.

As the immunoreactive epitopes within the vaccinated appeared to generally cluster in two regions (Figure 2), we defined these regions as Vaccination Cluster 1 (590–730aa; VC1) and Vaccination Cluster 2 (905–1115aa; VC2). Fisher's exact testing found there were no significant differences between the 7 of 26 (26.9%) epitopes located within VC1 that were immunoreactive in the vaccinated compared to the 4 of 30 (13.3%) in the naïve (in domain (%): 26.9% *vs*. 13.3%; *P* = 0.3130; Table 2) and 4 of 29 (13.8%) in the naturally infected (in domain (%): 26.9% *vs*. 13.8%; *P* = 0.3153; Table 2). In contrast, the 11 of 26 (42.3%) immunoreactive epitopes in the vaccinated that were located within VC2 were statistically significantly more than the 5 of 30 (16.7%) in the naïve (in domain (%): 42.3% *vs*. 16.7%; *P* = 0.0426; Table 2) and the 4 of 29 (13.8%) in the naturally infected (in domain (%): 42.3% *vs*. 13.8%; *P* = 0.0321; Table 2). When combined, 18 of 26 (69.2%) immunoreactive epitopes in the vaccinated were located within either VC1 or VC2, which was statistically significantly more than the 9 of 30 (30.0%) in the naïve (in domain (%): 69.2% *vs*. 30.0%; *P* = 0.0068; Table 2) and the 8 of 29 (27.6%) in the naturally infected (in domain (%): 69.2% *vs*. 27.6%; *P* = 0.0029; Table 2). There was no significant difference in the number of immunoreactive epitopes located within VC1, VC2 or when combined between the naïve and naturally infected (*P* > 0.9999 in all cases; Table 2). These data demonstrate that vaccination significantly altered the localisation of immunoreactive epitopes on the SARS‐CoV‐2 S‐protein, causing them to cluster predominantly in two specific regions.

Taken together, this study established that the most immunoreactive epitopes varied following vaccination and subsequent natural infection, shifting from HLA‐A in the naïve to HLA‐B in the naturally infected. Furthermore, although there was consistency between specific immunoreactive epitopes in naïve and vaccinated donors, vaccination significantly altered the localisation of immunoreactive epitopes, promoting epitopes that clustered within two extra‐domanial regions, while subsequent natural infection generally promoted novel epitopes.

## Discussion

In this study, we analysed the immunoreactivity of SARS‐CoV‐2 S‐protein peptide epitopes in donors across multiple kinetic timepoints from SARS‐CoV‐2 S‐protein naïve, following AstraZeneca double homologous vaccination, and subsequent natural exposure to SARS‐CoV‐2. The results of this study suggested the most immunoreactive SARS‐CoV‐2 S‐protein epitopes varied following vaccination and subsequent natural infection. Immunoreactive epitopes in the naïve were predominantly associated with HLA‐A, with nearly half clustering in the N‐terminal domain. After vaccination, there was a shift in the localisation of immunoreactive epitopes to two extra‐domanial regions. Subsequent natural infection induced novel epitopes that were dispersed across the antigen and primarily associated with HLA‐B, specifically the B7 supertype.

The immunoreactivity of SARS‐CoV‐2 S‐protein T‐cell epitopes has been extensively studied,^43^, ^57^ and many well‐characterised epitopes have been identified as immunoreactive across naïve, vaccinated and naturally infected donors (Supplementary table 3). Nevertheless, herein, we report immunoreactivity in several epitopes in immune status groups for the first time. For example, we report the A*02:01 epitope GLTVLPPLL was immunoreactive in a naïve donor, whereas previously this epitope has only been found to be immunoreactive following natural infection.^43^ In other epitopes, we report immunoreactivity which is in agreement with some of the literature. For example, we report the A*02:01 epitope VLNDILSRL was immunoreactive in a naïve and vaccinated donor, but we found no immunoreactivity following natural infection. Studies have reported the immunogenicity of VLNDILSRL in naïve individuals,^58^, ^59^ others have not,^60^ while some studies have reported immunogenicity only in naturally infected individuals,^31^ or reported immunogenicity only following certain immunisation strategies.^44^, ^61^ Indeed, many of the epitopes we identified as immunoreactive have inconsistent findings reported in IEDB (Supplementary table 3). Such inconsistencies are frequent in human studies because of the significant environmental and genetic variation inherent in human donors. These inconsistencies may also stem from the influence and interaction of underreported technical variation, such as during PBMC collection, cryopreservation, thawing and culture.^62^ The variation in donor HLA types, available PBMCs and the relatively small sample size (≤ 10 donors per group) of this study is also likely to introduce variability. Larger studies with more uniformly sampled donors would help confirm and expand the observation of this study.

We found there was a statistically significant increase in the number of HLA‐B7 supertype epitopes that were immunogenic following natural infection. HLA‐B genes have a reported strong association with viral infections,^63^, ^64^ including in COVID‐19,^65^ especially the B7 supertype allele HLA‐B*15:01, which has been associated with asymptomatic SARS‐CoV‐2 infections,^66^ and HLA‐B*07:02, which is associated with a high degree of pre‐existing cross‐reactive memory T cells.^67^ Nevertheless, it remains unclear how increasing HLA‐B7 allele immunogenicity influences patient outcomes, as HLA‐B7 supertype alleles have been associated with increased disease susceptibility.^68^, ^69^ It is also unclear why peptide epitopes associated with HLA‐A genes would be prominently immunoreactive in the naïve. There is significant peptide overlap amongst common S‐protein epitopes and endogenous tumour‐associated epitopes,^70^ which may be activated during healthy immune homeostasis, and core amino acid anchors of epitopes immunoreactive in the naive have found broad anchor homology in seasonal coronavirus. It remains unclear whether this pre‐existing immunity is irrelevant or related to beneficial or detrimental patient outcomes.^71^ Our analysis revealed that sequence homology was not correlated with epitope immunogenicity. This finding aligns with existing literature, which indicates that CD4^+^ and CD8^+^ T‐cell responses in convalescent COVID‐19 patients or recipients of the COVID‐19 mRNA vaccines were not significantly impacted by mutations present in SARS‐CoV‐2 variants.^72^ However, given the tens of thousands of possible epitopes available within the S‐protein, more extensive studies are needed to definitively determine the relationship between sequence homology, viral variants, vaccines and epitope immunogenicity.

Our study identified the localisation of immunoreactive epitopes in two extra‐domanial regions, between 590–730aa and 905–1115aa of the S‐protein following vaccination. Other studies have shown dynamic changes in epitope localisation amongst donors with varying immune statuses. For example, one study found that individuals previously infected with SARS‐CoV‐2 develop more distinct T‐cell immune memory compared to those who are only vaccinated.^73^ Another study observed that, in naïve patients, both the C‐ and N‐terminal regions of the ORF1 protein contain fewer T‐cell epitopes, and in agreement with this study, reported similar epitope localisation in convalescent and naïve patients.^60^ It is unclear why such localisation of T‐cell epitopes would occur in donors of various immune statuses.

The only post‐vaccination samples assessed in this study were PBMCs collected from donors following a double homologous AstraZeneca ChAdOx1 nCoV‐19 (AZ) vaccination regimen. The AZ vaccine has been found to induce potent CD4^+^ and CD8^+^ T‐cell responses.^74^, ^75^ Heterologous boosting with other vaccines, such as the BNT162b2 Pfizer‐BioNTech (Pfizer) vaccine, may induce stronger immune responses than a homologous regimen.^18^ Investigating the reactivity of peptide epitopes across varying homologous and heterologous vaccine regimens may provide further insight into improving vaccine‐induced T‐cell immunogenicity against SARS‐CoV‐2. Post‐vaccination and post‐infection samples were collected between 1 and 4 months following convalescence, and therefore, the T‐cell reactivity observed in this study was more likely associated with long‐term immune memory cells.^76^ However, flow‐cytometry reactive cell phenotyping would be required to identify which cells are responsible for the immunoreactivity. It is possible that investigating the kinetics of acute phase peptide epitope immunoreactivity and subsequent long‐term memory formation may provide insight into the development of long‐term cellular immunity.

The *in vivo* development of a T‐cell peptide epitope immunodominance hierarchy is a complex process, which remains incompletely understood.^77^ However, several aspects of the formation of immunodominance, such as the relationship between immunogenicity and MHC binding affinity, are relatively well established. Indeed, the binding affinity of peptide epitopes to variable MHC alleles is described as the most selective stage of the formation of an epitope immunodominance hierarchy,^33^ and a binding affinity threshold of 500 nm for peptide–MHC interactions has been experimentally established as necessary to initiate T‐cell immunity.^78^ We found the Immune Epitope Database (IEDB) HLA‐peptide binding score did identify peptide epitopes found to be highly immunoreactive in convalescent COVID‐19 patients.^43^ However, the predictive binding affinity of a putative peptide epitope for a given HLA molecule should not be used as the sole predictor of immunodominance.^79^, ^80^ Variables, including previous exposure to homologous epitopes, antigen abundance following vaccination and infection, antigen processing, peptide‐HLA‐binding competition and T‐cell receptor (TCR) repertoire, can all influence the immunodominance hierarchy.^77^ We found abundant cross HLA‐binding affinities in our prioritised peptide epitopes (Supplementary figure 2). To investigate how the cross‐HLA‐binding affinities of peptides predicted from the SARS‐CoV‐2 S‐protein impact epitope immunogenicity, a validated immunodominance hierarchy of S‐protein epitopes would need to be constructed across variably HLA‐matched donors and compared to HLA‐peptide binding scores.

Variance in peptide epitope immunogenicity between post‐vaccinated and ‐infected individuals can be partially explained by exposure to viral variants carrying epitope mutations within theS‐protein. We found variant‐defining mutations crossed several of our peptide epitopes, with the Omicron variant carrying the largest number of S‐protein nonsynonymous mutations, mostly within the receptor binding domain (Supplementary figure 3). By late 2023, the most dominant variant circulating globally was Omicron, and due to its global distribution, many sub‐variants are now circulating, each with defining mutations.^81^ Interestingly, Omicron sub‐variants are undergoing convergent evolution, as several areas in the RBD have appeared as mutational hotspots.^82^ While the cause remains unknown, it is likely both humoral and cellular immunity are applying selective pressure to these strains. It is likely that genomic surveillance of viral lineages amongst PBMC donors would provide significant insight into how variants can influence epitope immunogenicity, and how this may relate to the variant evolution.^83^

In summary, herein we have investigated the immunogenicity of 170 immunodominant peptide epitopes from the S‐protein of SARS‐CoV‐2 in pre‐vaccinated, post‐vaccinated and post‐infected individuals. Our investigation revealed that immunoreactivity was not confined to a select few immunodominant epitopes; instead, it was widely distributed amongst numerous epitopes. Immunoreactive epitopes in the naïve were predominantly associated with HLA‐A, which shifted to primary HLA‐B following natural infection. Vaccination promoted epitopes that were immunoreactive in the naïve, but primarily within two regions, and natural infection generally promoted novel epitopes that were dispersed across the S‐protein antigen.

## Methods

### Peptides

#### CD8^+^ T‐cell epitope prediction and selection

Over 7500 CD8^+^ T‐cell peptide epitope–HLA allele combinations with an HLA‐binding score > 0.2 were predicted from the S‐protein of Wuhan reference strain of SARS‐CoV‐2 (GenBank: YP_009724390.1) using the Immune Epitope Database (IEDB) NetMHCpan EL 4.1 algorithm^49^ (Supplementary figure 1). A prioritised list of 145 peptides was defined by sorting epitopes into affinity of binding to HLA‐A2, HLA‐A3/11, HLA‐A24, HLA‐B7, and HLA‐B8 Class I supertypes, ordering by the sum of the response as reported from Tarke *et al*.^43^ and, subsequently, the IEDB predicted binding score (Supplementary table 1). Other peptides reported in the literature to be immunoreactive were also included^21^, ^41^, ^51^ (Supplementary table 1).

#### CD4^+^ T‐cell epitope selection

Twenty‐five CD4^+^ T‐cell S‐protein peptide epitopes reported as immunogenic following exposure to SARS‐CoV‐2 were selected from the literature^43^, ^46^, ^52^ (Supplementary table 1).

#### *In silico* predicted CD8^+^ peptide epitope cross‐HLA‐binding affinity analysis

The capacity of predicted CD8^+^ T‐cell peptide epitopes to bind degenerately to multiple HLA alleles (cross‐HLA‐binding affinity) was determined using the Immune Epitope Database (IEDB) NetMHCpan EL 4.1 algorithm^49^ queried to predict a binding score of each peptide across 27 common alleles within the HLA‐A1, A2, A3/11, A24, A3/01, B7, B8, B44, B58 and B62 HLA‐supertypes (Supplementary figure 2).

#### *In silico* CD4^+^ and CD8^+^ T‐cell epitope homology analysis

Variant‐defining consensus nonsynonymous mutations in SARS‐CoV‐2 S‐protein relative to the Wuhan consensus sequence were provided by CoVariants^84^ (accessed October 2023) using data from the *Global Initiative on Sharing All Influenza Data* (GISAID). Each peptide (Supplementary figure 3) and the Alpha (B.1.1.7; GenBank: QWE88920.1), Beta (B.1.351; GenBank: QRN78347.1), Gamma (B.1.1.28.1; GenBank: QVE55289.1), Delta (B.1.617.2; GenBank: QWK65230.1) and Omicron (B.1.1.529; GenBank: UFO69279.1) strains, and SARS‐CoV1 (YP_009825051.1), MERS (GenBank: YP_009047204.1), CovNL63 (GenBank: YP_003767.1), CoV‐229E (GenBank: AAK32191.1), Cov43 (GenBank: QXL74886.1) and CovHKU1 (GenBank: YP_173238.1) species of coronavirus were aligned to the SARS‐CoV‐2 Wuhan strain (GenBank: YP_009724390.1) using the Multiple Sequence Comparison by Log‐Expectation (MUSCLE; European Bioinformatics Institute) alignment tool with standard settings.^85^

#### Peptides

T‐cell peptide epitopes from SARS‐CoV‐2 S‐protein were synthesised at 95% purity (Mimotopes, Melbourne, Australia) and resuspended in dimethyl sulfoxide (DMSO) at a concentration of 20 mg mL^−1^.^86^ A positive control CEF peptide pool representing well‐characterised CD8^+^ T‐cell epitopes from Influenza virus, Epstein–Barr virus and Cytomegalovirus (CEF (HLA Class I Control) Peptide Pool) was purchased commercially (Stem Cell Technologies).

### Samples

#### Ethical approval

This study was performed according to the principles of the Declaration of Helsinki. Ethics approval to undertake the research was obtained from QIMR Berghofer Medical Research Institute Human Research Ethics Committee (HREC: P2282). Informed consent was obtained from all participants. The inclusion criteria for the study were that participants were over the age of 18 and were well and able to donate in adherence with Queensland Health policies. All methods were performed in accordance with institutional guidelines and regulations.

#### Samples

PBMCs and sera were collected from 11 individuals at three timepoints: (1) Pre‐2019 (SARS‐CoV‐2‐naïve), (2) Four weeks post double homologous AstraZeneca™ (ChAdOx1 nCoV‐19) vaccination (vaccinated) and (3) 4 weeks post subsequent SARS‐CoV‐2 infection (naturally infected). Information about which variant each donor was infected with was not available, but the dominant variant circulating during the sampling period was the original Wuhan strain, followed by the Delta and Omicron variants. There were no mortality or severe outcomes (i.e. hospitalisation) during convalescence for all naturally infected donors. Human leukocyte antigen (HLA) typing was performed by AlloSeq Tx17 (CareDx Pty Ltd, Fremantle, Australia) (Supplementary table 2). PBMCs were isolated by standard density gradient centrifugation and cryopreserved in 90% FBS/10% DMSO as previously described.^53^ PBMCs were thawed at 37°C, rested for 18 h at 2 × 10^6^ cells mL^−1^ in RPMI‐1640 supplemented with 10% heat‐inactivated AB human serum (Sigma‐Aldrich), 100 U mL^−1^ penicillin/streptomycin (Thermo Fisher Scientific), 2 mm GlutaMAX (Thermo Fisher Scientific), 10 mm HEPES (Thermo Fisher Scientific) (R10 Media), at 37°C and 10% CO_2_.

#### Detection of SARS‐CoV‐2 antibodies

Sera were collected from serum‐separating tubes following centrifugation as previously described.^87^ Serum antibody titres were analysed on a cobas e411 (Roche Diagnostics) and Abbott Architect i4000SR (Abbott Diagnostics) analyser using their respective assays as described by the manufacturer. The Abbott SARS‐CoV‐2 IgG assay detected IgG antibodies to the receptor binding domain (RBD) of the S1 subunit of the S‐protein of SARS‐CoV‐2. The Roche assay detected both IgM and IgG antibodies reactive to the nucleocapsid protein (NCAP) of SARS‐CoV‐2. Both assays report their respective quantitative signal (QS).

#### T‐cell assays

PBMCs were stimulated in 50 μL R10 media in 96‐well U‐bottom plates for 6 h before cells were lysed in MagMAX Lysis Buffer, as previously described.^56^ The number of PBMCs stimulated was normalised across kinetic timepoints but varied between donors (3.4–11.5 × 10^5^ PBMCs/Stimulation; Supplementary table 2). Predicted SARS‐CoV‐2 T‐cell peptides and CEF peptide pool at 2 μg mL^−1^ were tested alongside 50 ng mL^−1^ PMA, 1000 ng mL^−1^ Ionomycin mitogen positive control and a media‐only negative control.

### Reverse transcription‐quantitative PCR

RNA was extracted and converted to cDNA using our ‘High‐Throughput Screening (HTS) optimised protocol’ as previously described.^56^ Briefly, RNA was isolated using a MagMAX™ *mirVana*™ Total RNA Isolation Kit (Applied Biosystems) and converted to cDNA with SuperScript™ IV First‐Strand Synthesis System (Thermo Fisher) following the manufacturer's instructions, except that all reagents were used at 25% of the volume recommended by the manufacturer, respectively; and the Superscript™ IV reverse transcriptase enzyme was used at 5 U μL^−1^ RNA. Due to the variable numbers of stimulated PBMCs across donors (Supplementary table 2), relative quantification was used for qPCR, as previously described.^88^ Briefly, the fold‐change expression of *Interferon gamma* (IFN‐γ) were determined relative to the expression of the reference gene *Ribosomal protein L13a* (*RPL13a*). Fold change was normalised relative to the negative control (media only) stimulation. *IFN‐γ*, and *RPL13a* specific desalt‐grade (Sigma‐Aldrich) previously optimised primers,^53^ obtained from PrimerBank™^89^ were used at 500 nm using ssoAdvanced™ Universal SYBR® Green Master‐Mix (Bio‐Rad). All reactions were run in technical triplicate in accordance with MIQE guidelines^88^ at 5 μL total volume with 1 μL of reverse transcription eluent diluted 1:4 in Ultra‐Pure™ H_2_O (Invitrogen). Data were acquired using a QuantStudio5 Real‐Time PCR system running QuantStudio Design and Analysis Software (v1.4.3; Applied Biosystems).

### Data analysis

To examine the immunoreactivity of peptide epitopes, stimulations were classified as either positive or negative, and these categorical data were tested with Fisher's exact testing. Further categorical data, including epitope homology (Homologous *vs*. Not Homologous) and immunoreactive epitope localisation (within region *vs*. outside region), were also tested with a Fisher's exact test. Kinetic data examining the strength of immunoreactivity of peptide epitopes across donor‐matched timepoints were tested with a non‐parametric Kruskal–Wallis test, followed by Dunn's corrected multiple comparisons testing. GraphPad Prism version 10.2.0 (GraphPad Software) was used, and in all cases, *P‐*values < 0.05 were considered statistically significant.

## Conflict of interest

The authors declare no conflict of interest.

## Author contributions

**Daniel J Browne:** Conceptualization; formal analysis; methodology; writing – original draft; writing – review and editing. **Pauline Crooks:** Methodology; writing – review and editing. **Corey Smith:** Conceptualization; supervision; writing – review and editing. **Denise L Doolan:** Conceptualization; formal analysis; funding acquisition; project administration; supervision; writing – original draft.

## Supporting information


Supplementary figures 1–4
Supplementary tables 1–7

## Data Availability

The original contributions presented in the study are included the article and Supplementary material. Further inquiries can be directed to the corresponding author.
