# Supplementary material for: Differential reactivity of SARS‐CoV‐2 S‐protein T‐cell epitopes in vaccinated versus naturally infected individuals
Source: Clin Transl Immunology. 2025 May 6;14(5):e70031. doi: 10.1002/cti2.70031 (PMC12056234; doi:10.1002/cti2.70031)
Supplement: Supplementary file 1 — Supplementary figures 1–4 Supplementary tables 1–7 [file CTI2-14-e70031-s001.docx]

***Supplementary information***

| 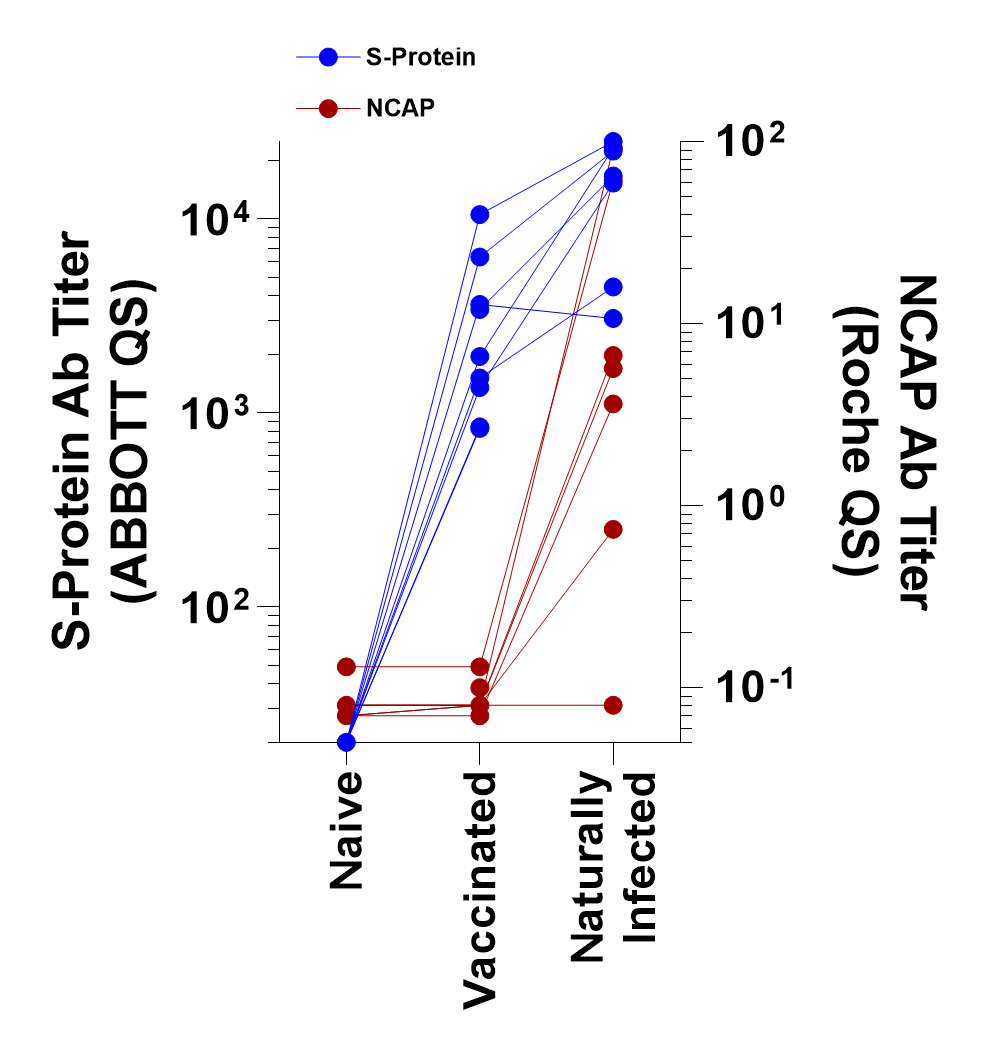 |
| --- |
| **Supplementary figure 1. Donor Serology.** Antibody (Ab) titres of Donors 1 to 11 expressed as a quantitative signal (QS) specific to the SARS-COV-2 Spike Protein (S-Protein; Blue points) and nucleocapsid (NCAP; red points) as quantified with the Abbott SARS-COV-2 IgG assay and Roche IgG and IgM assays, respectively. |


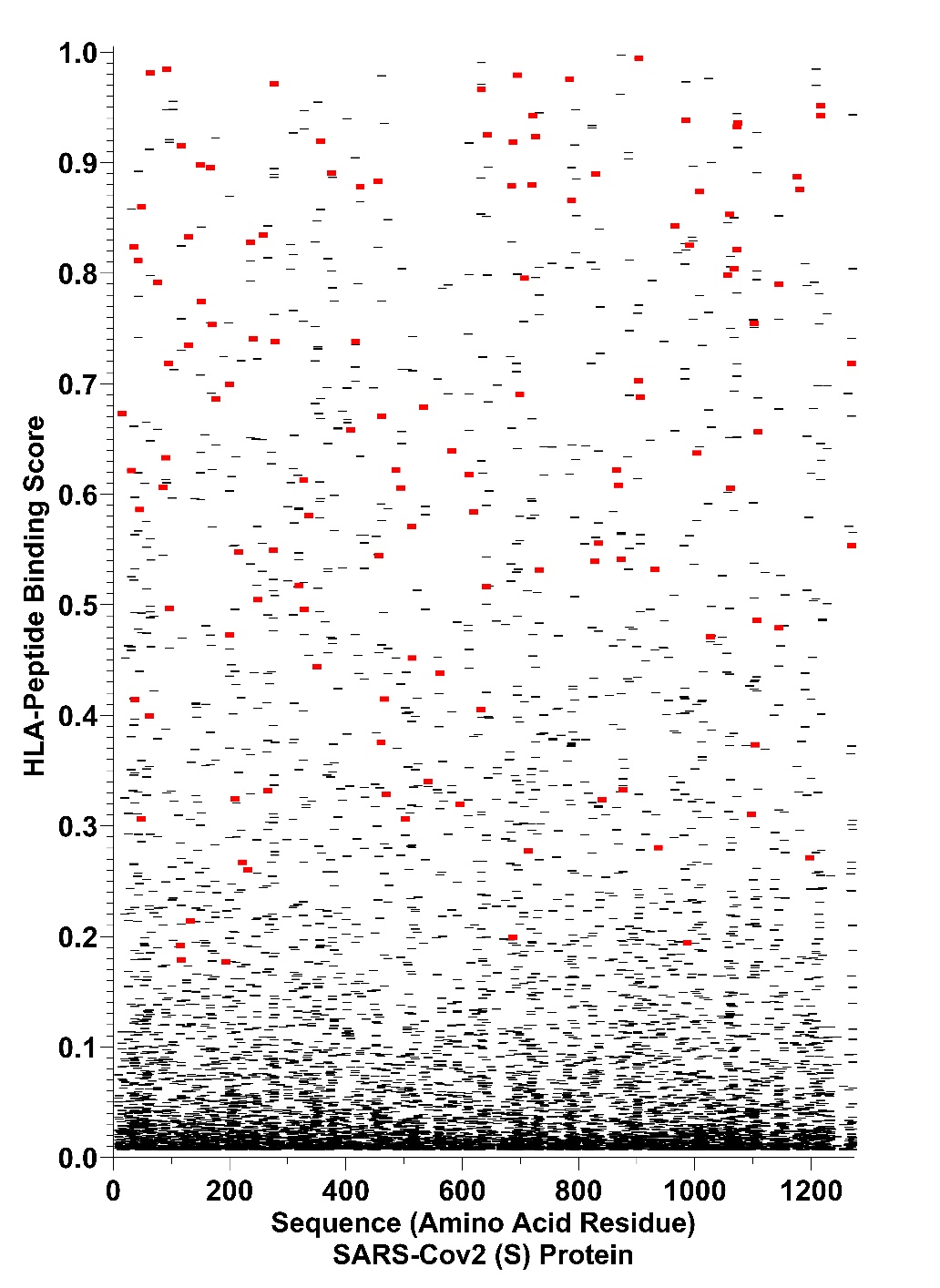


**Supplementary figure 2. Selection of a putative list of CD8^+^ T cell** SARS-COV-2 **S-protein peptide epitopes from IEDB predicted epitopes.** A putative list of MHC Class I SARS-COV-2 S-protein epitopes predicted to bind with high affinity to any allelic variant of the HLA-A or -B genes contained 7749 MHC Class I peptide epitopes identified with a HLA-peptide binding score > 0.007 were distributed across the sequence of the SARS-COV-2 S-protein (Black lines). A final list of 170 peptides were selected (139 Class I shown; Red lines) prioritised by binding to HLA supertype A2, A3/A11, A24, B7 and B8, and further prioritised by identification as immunoreactive in the literature ^1^.


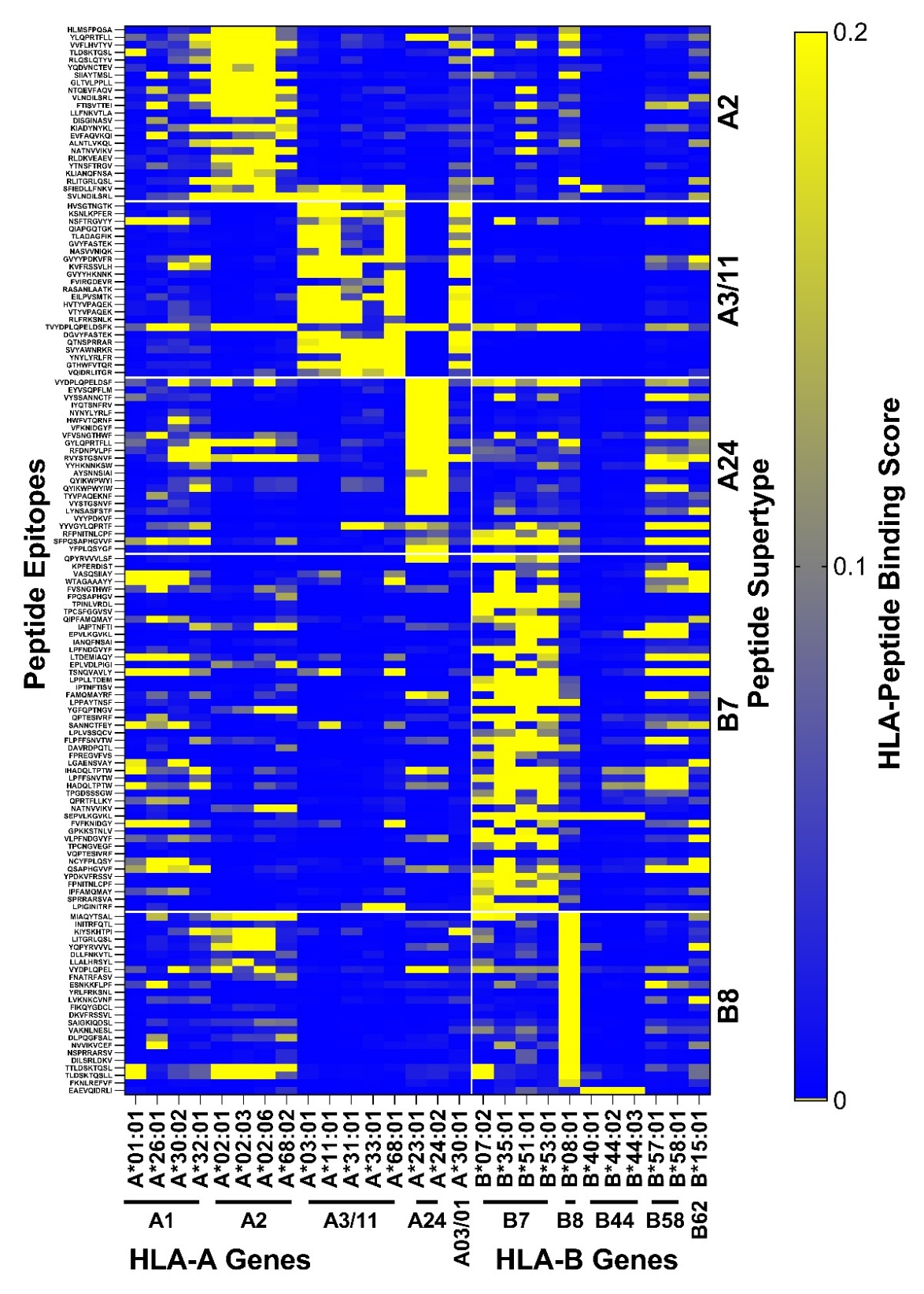


**Supplementary figure 3. Predicted CD8^+^ peptide epitope HLA affinity.** A heatmap visualisation of the *in silico* cross-HLA binding affinity of our prioritized CD8^+^ peptide list by querying IEDB to predict a binding score of each peptide across 27 common HLA alleles. Relatively strong peptide-HLA interactions (HLA binding scores >0.2) were typically predicted between most HLA alleles within a supertype. Heatmap organised with SARS-COV-2 S-protein peptide epitopes (y-axis; left) sorted into HLA A2, A3/11, A24, B7, B8, supertype classifications (y-axis; right). Yellow is a relatively high binding score while blue is a relatively low binding score.


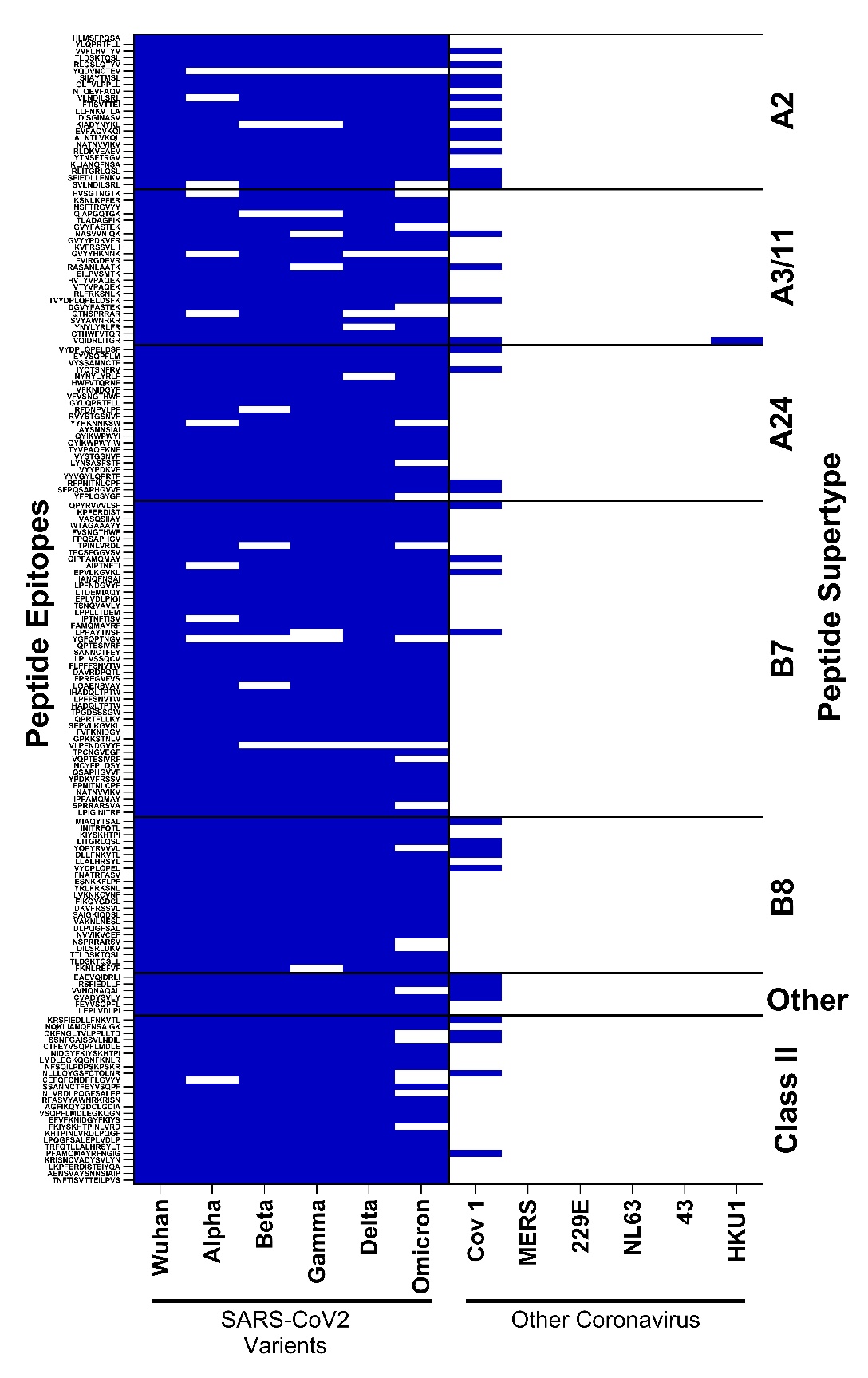


**Supplementary figure 4. Homology of peptide epitopes to clinically relevant circulating** SARS-COV-2 **variants and other endemic coronaviruses.** A heatmap visualisation of peptide homology to coronavirus spike-protein amino acid sequences where blue is 100% homology and white is at least one synonymous mutation. Heatmap is organised with SARS-COV-2 S-protein peptide epitopes (*y*-axis; left) sorted into HLA-A2, -A3/11, -A24, -B7, -B8, other, and Class II supertype classifications (*y*-axis; right).

***Supplementary tables***

**Supplementary table 1. SARS-COV-2 S-protein T cell peptide epitopes**

Shown are the order of the epitopes (Number: N^o^) amino acid 1-letter symbols (Sequence), the predicted Human Leukocyte Antigen (HLA) allele with the highest affinity for the sequence, the HLA-Supertype of the allele, the amino acid number along the S-protein sequence that the epitope beings (Start), the amino acid number along the S-protein sequence that the epitope ends (End), the IEDB predicted binding score (Binding Score), and the sum of the response as identified from Tarke *et al*, 2021 ^2^ (Sum of Response), which immune status this epitope was identified as immunoreactive in this study (Positive Immune Status). Peptides sequentially ordered by HLA supertype, Sum of Response, then Binding Score. N/I – Natural Infection.

| **No.** | **Sequence** | **Allele** | **HLA supertype** | **Start** | **End** | **Binding score** | **Sum of response** | **Positive immune status** |
| --- | --- | --- | --- | --- | --- | --- | --- | --- |
| 1 | HLMSFPQSA | A*02:01 | A2 | 1048 | 1056 | 0.798 | 1011 | Naïve |
| 2 | YLQPRTFLL | A*02:01 | A2 | 269 | 277 | 0.971 | 404 | Vaccinated |
| 3 | VVFLHVTYV | A*02:03 | A2 | 1060 | 1068 | 0.804 | 365 | N/I |
| 4 | TLDSKTQSL | A*02:01 | A2 | 109 | 117 | 0.915 | 296 |  |
| 5 | RLQSLQTYV | A*02:01 | A2 | 1000 | 1008 | 0.874 | 264 | Naïve & Vaccinated |
| 6 | YQDVNCTEV | A*02:06 | A2 | 612 | 620 | 0.584 | 243 | Vaccinated & N/I |
| 7 | SIIAYTMSL | A*02:06 | A2 | 691 | 699 | 0.69 | 165 | Vaccinated |
| 8 | GLTVLPPLL | A*02:01 | A2 | 857 | 865 | 0.622 | 151 | Naïve |
| 9 | NTQEVFAQV | A*68:02 | A2 | 777 | 785 | 0.976 |  |  |
| 10 | VLNDILSRL | A*02:01 | A2 | 976 | 984 | 0.938 |  | Naïve & Vaccinated |
| 11 | FTISVTTEI | A*68:02 | A2 | 718 | 726 | 0.924 |  |  |
| 12 | LLFNKVTLA | A*02:03 | A2 | 821 | 829 | 0.89 |  |  |
| 13 | DISGINASV | A*68:02 | A2 | 1168 | 1176 | 0.887 |  | N/I |
| 14 | KIADYNYKL | A*02:06 | A2 | 417 | 425 | 0.879 |  | Naïve, & Vaccinated |
| 15 | EVFAQVKQI | A*68:02 | A2 | 780 | 788 | 0.866 |  |  |
| 16 | ALNTLVKQL | A*02:03 | A2 | 958 | 966 | 0.843 |  | Naïve |
| 17 | NATNVVIKV | A*68:02 | A2 | 122 | 130 | 0.833 |  |  |
| 18 | RLDKVEAEV | A*02:01 | A2 | 983 | 991 | 0.825 |  |  |
| 19 | YTNSFTRGV | A*68:02 | A2 | 28 | 36 | 0.824 |  |  |
| 20 | KLIANQFNSA | A*02:03 | A2 | 920 | 929 |  |  |  |
| 21 | RLITGRLQSL | A*02:03 | A2 | 995 | 1004 |  |  |  |
| 22 | SFIEDLLFNKV | A*02:06 | A2 | 817 | 826 |  |  |  |
| 23 | SVLNDILSRL | A*02:03 | A2 | 974 | 983 |  |  |  |
| 24 | HVSGTNGTK | A*68:01 | A3/A11 | 69 | 78 | 0.792 | 972 |  |
| 25 | KSNLKPFER | A*11:01 | A3/A11 | 458 | 466 | 0.415 | 701 |  |
| 26 | NSFTRGVYY | A*68:01 | A3/A11 | 30 | 38 | 0.414 | 600 | Naïve |
| 27 | QIAPGQTGK | A*11:01 | A3/A11 | 409 | 417 | 0.738 | 518 |  |
| 28 | TLADAGFIK | A*03:01 | A3/A11 | 827 | 835 | 0.556 | 398 |  |
| 29 | GVYFASTEK | A*68:01 | A3/A11 | 89 | 97 | 0.497 | 340 | N/I |
| 30 | NASVVNIQK | A*68:01 | A3/A11 | 1173 | 1181 | 0.876 | 326 |  |
| 31 | GVYYPDKVFR | A*31:01 | A3/A11 | 35 | 44 | 0.811 | 314 | Naïve |
| 32 | KVFRSSVLH | A*03:01 | A3/A11 | 41 | 49 | 0.86 | 272 |  |
| 33 | GVYYHKNNK | A*03:01 | A3/A11 | 142 | 150 | 0.898 | 270 | Naïve |
| 34 | FVIRGDEVR | A*68:01 | A3/A11 | 400 | 408 | 0.658 | 269 |  |
| 35 | RASANLAATK | A*03:01 | A3/A11 | 1019 | 1028 | 0.471 | 268 | Vaccinated |
| 36 | EILPVSMTK | A*03:01 | A3/A11 | 725 | 733 | 0.532 | 253 |  |
| 37 | HVTYVPAQEK | A*68:01 | A3/A11 | 1064 | 1073 | 0.821 | 250 | Vaccinated |
| 38 | VTYVPAQEK | A*03:01 | A3/A11 | 1065 | 1073 | 0.932 | 235 |  |
| 39 | RLFRKSNLK | A*11:01 | A3/A11 | 454 | 462 | 0.671 | 233 |  |
| 40 | TVYDPLQPELDSFK | A*03:01 | A3/A11 | 1135 | 1149 |  | 225 |  |
| 41 | DGVYFASTEK | A*68:01 | A3/A11 | 88 | 97 | 0.719 | 205 | Naïve |
| 42 | QTNSPRRAR | A*31:01 | A3/A11 | 677 | 685 | 0.879 | 200 | N/I |
| 43 | SVYAWNRKR | A*31:01 | A3/A11 | 349 | 357 | 0.92 | 190 | Naïve, & Vaccinated |
| 44 | YNYLYRLFR | A*33:01 | A3/A11 | 449 | 457 | 0.545 | 170 |  |
| 45 | GTHWFVTQR | A*03:01 | A3/A11 | 1099 | 1107 | 0.486 | 164 |  |
| 46 | VQIDRLITGR | A*68:01 | A3/A11 | 1000 | 1009 |  |  |  |
| 47 | VYDPLQPELDSF | A*23:01 | A24 | 1137 | 1145 | 0.79 | 2244 |  |
| 48 | EYVSQPFLM | A*24:02 | A24 | 169 | 177 | 0.686 | 815 | Naïve |
| 49 | VYSSANNCTF | A*24:02 | A24 | 159 | 168 | 0.895 | 719 | Naïve, & Vaccinated |
| 50 | IYQTSNFRV | A*24:02 | A24 | 312 | 320 | 0.518 | 710 | Naïve |
| 51 | NYNYLYRLF | A*24:02 | A24 | 448 | 456 | 0.883 | 606 |  |
| 52 | HWFVTQRNF | A*24:02 | A24 | 1101 | 1109 | 0.656 | 551 | Vaccinated & N/I |
| 53 | VFKNIDGYF | A*24:02 | A24 | 193 | 201 | 0.473 | 467 |  |
| 54 | VFVSNGTHWF | A*24:02 | A24 | 1094 | 1102 | 0.755 | 465 | N/I |
| 55 | GYLQPRTFLL | A*24:02 | A24 | 268 | 277 | 0.549 | 378 |  |
| 56 | RFDNPVLPF | A*24:02 | A24 | 78 | 86 | 0.606 | 365 | N/I |
| 57 | RVYSTGSNVF | A*24:02 | A24 | 634 | 643 | 0.516 | 349 | Naïve & Vaccinated |
| 58 | YYHKNNKSW | A*23:01 | A24 | 144 | 152 | 0.774 | 348 | Naïve |
| 59 | AYSNNSIAI | A*24:02 | A24 | 706 | 714 | 0.277 | 278 |  |
| 60 | QYIKWPWYI | A*24:02 | A24 | 1208 | 1216 | 0.952 | 214 |  |
| 61 | QYIKWPWYIW | A*23:01 | A24 | 1208 | 1217 | 0.943 | 193 |  |
| 62 | TYVPAQEKNF | A*24:02 | A24 | 1066 | 1075 | 0.936 |  | Naïve |
| 63 | VYSTGSNVF | A*24:02 | A24 | 635 | 643 | 0.925 |  |  |
| 64 | LYNSASFSTF | A*24:02 | A24 | 368 | 377 | 0.89 |  |  |
| 65 | VYYPDKVF | A*24:02 | A24 | 34 | 43 |  | 167 | Naïve |
| 66 | YYVGYLQPRTF | A*24:02 | A24 | 264 | 275 |  | 1452 |  |
| 67 | RFPNITNLCPF | A*24:02 | A24 | 327 | 338 |  | 539 |  |
| 68 | SFPQSAPHGVVF | A*24:02 | A24 | 1050 | 1062 |  | 234 | Vaccinated |
| 69 | YFPLQSYGF | A*29:02 | A24 | 489 | 497 |  |  | N/I |
| 70 | QPYRVVVLSF | B*07:02 | B7 | 506 | 515 | 0.452 | 3589 |  |
| 71 | KPFERDIST | B*07:02 | B7 | 462 | 470 | 0.329 | 2921 | Naïve |
| 72 | VASQSIIAY | B*35:01 | B7 | 687 | 695 | 0.979 | 1444 |  |
| 73 | WTAGAAAYY | B*35:01 | B7 | 258 | 266 | 0.332 | 1401 |  |
| 74 | FVSNGTHWF | B*35:01 | B7 | 1095 | 1103 | 0.373 | 436 |  |
| 75 | FPQSAPHGV | B*51:01 | B7 | 1052 | 1060 | 0.853 | 413 |  |
| 76 | TPINLVRDL | B*07:02 | B7 | 208 | 216 | 0.548 | 406 |  |
| 77 | TPCSFGGVSV | B*07:02 | B7 | 588 | 597 | 0.319 | 405 |  |
| 78 | QIPFAMQMAY | B*35:01 | B7 | 895 | 904 | 0.703 | 404 | N/I |
| 79 | IAIPTNFTI | B*51:01 | B7 | 712 | 720 | 0.88 | 355 |  |
| 80 | EPVLKGVKL | B*07:02 | B7 | 1262 | 1270 | 0.553 | 313 |  |
| 81 | IANQFNSAI | B*51:01 | B7 | 923 | 931 | 0.532 | 307 |  |
| 82 | LPFNDGVYF | B*35:01 | B7 | 84 | 92 | 0.985 | 299 |  |
| 83 | LTDEMIAQY | B*35:01 | B7 | 865 | 873 | 0.541 | 294 | N/I |
| 84 | EPLVDLPIGI | B*51:01 | B7 | 224 | 233 | 0.261 | 294 |  |
| 85 | TSNQVAVLY | B*35:01 | B7 | 604 | 612 | 0.618 | 278 | Vaccinated & N/I |
| 86 | LPPLLTDEM | B*35:01 | B7 | 861 | 869 | 0.608 | 264 | Vaccinated & N/I |
| 87 | IPTNFTISV | B*51:01 | B7 | 714 | 722 | 0.943 | 254 | Naïve, &  Vaccinated |
| 88 | FAMQMAYRF | B*35:01 | B7 | 898 | 906 | 0.688 | 236 |  |
| 89 | LPPAYTNSF | B*53:01 | B7 | 24 | 32 | 0.622 | 232 |  |
| 90 | YGFQPTNGV | B*51:01 | B7 | 495 | 503 | 0.306 | 208 |  |
| 91 | QPTESIVRF | B*51:01 | B7 | 321 | 329 | 0.496 | 193 | N/I |
| 92 | SANNCTFEY | B*35:01 | B7 | 162 | 170 | 0.753 | 192 | Vaccinated |
| 93 | LPLVSSQCV | B*51:01 | B7 | 8 | 16 | 0.673 | 175 |  |
| 94 | FLPFFSNVTW | B*35:01 | B7 | 55 | 64 | 0.4 | 163 |  |
| 95 | DAVRDPQTL | B*51:01 | B7 | 574 | 582 | 0.639 | 157 | N/I |
| 96 | FPREGVFVS | B*35:01 | B7 | 1089 | 1097 | 0.31 | 156 |  |
| 97 | LGAENSVAY | B*35:01 | B7 | 699 | 707 | 0.795 | 125 |  |
| 98 | IHADQLTPTW | B*53:01 | B7 | 624 | 633 | 0.405 | 122 |  |
| 99 | LPFFSNVTW | B*53:01 | B7 | 56 | 64 | 0.981 |  |  |
| 100 | HADQLTPTW | B*53:01 | B7 | 625 | 633 | 0.966 |  |  |
| 101 | TPGDSSSGW | B*53:01 | B7 | 250 | 258 | 0.834 |  |  |
| 102 | QPRTFLLKY | B*35:01 | B7 | 271 | 279 | 0.738 |  | Vaccinated & N/I |
| 103 | SEPVLKGVKL | B*07:02 | B7 | 1261 | 1270 | 0.718 |  |  |
| 104 | FVFKNIDGY | B*35:01 | B7 | 192 | 200 | 0.7 |  |  |
| 105 | GPKKSTNLV | B*07:02 | B7 | 526 | 534 | 0.679 |  |  |
| 106 | VLPFNDGVYF | B*53:01 | B7 | 83 | 92 | 0.633 |  |  |
| 107 | TPCNGVEGF | B*35:01 | B7 | 478 | 486 | 0.622 |  |  |
| 108 | VQPTESIVRF | B*53:01 | B7 | 320 | 329 | 0.613 |  | Naïve & N/I |
| 109 | NCYFPLQSY | B*35:01 | B7 | 487 | 495 | 0.606 |  |  |
| 110 | QSAPHGVVF | B*35:01 | B7 | 1054 | 1062 | 0.606 |  |  |
| 111 | YPDKVFRSSV | B*07:02 | B7 | 38 | 47 | 0.586 |  |  |
| 112 | FPNITNLCPF | B*35:01 | B7 | 329 | 338 | 0.581 |  |  |
| 113 | LPFFSNVTWF | B*53:01 | B8 | 56 | 65 | 0.56 |  |  |
| 114 | IPFAMQMAY | B*35:01 | B7 | 896 | 904 | 0.994 |  | N/I |
| 115 | SPRRARSVA | B*07:02 | B7 | 680 | 688 | 0.919 |  |  |
| 116 | LPIGINITRF | B*35:01 | B7 | 229 | 238 | 0.828 |  |  |
| 117 | MIAQYTSAL | B*08:01 | B8 | 869 | 877 | 0.332 | 1025 |  |
| 118 | INITRFQTL | B*08:01 | B8 | 233 | 241 | 0.741 | 402 |  |
| 119 | KIYSKHTPI | B*08:01 | B8 | 202 | 210 | 0.325 | 298 | N/I |
| 120 | LITGRLQSL | B*08:01 | B8 | 996 | 1004 | 0.637 |  |  |
| 121 | YQPYRVVVL | B*08:01 | B8 | 505 | 513 | 0.571 |  |  |
| 122 | DLLFNKVTL | B*08:01 | B8 | 820 | 828 | 0.539 |  | N/I |
| 123 | LLALHRSYL | B*08:01 | B8 | 241 | 249 | 0.505 |  | N/I |
| 124 | VYDPLQPEL | B*08:01 | B8 | 1137 | 1145 | 0.479 |  |  |
| 125 | FNATRFASV | B*08:01 | B8 | 342 | 350 | 0.444 |  |  |
| 126 | ESNKKFLPF | B*08:01 | B8 | 554 | 562 | 0.438 |  |  |
| 127 | YRLFRKSNL | B*08:01 | B8 | 453 | 461 | 0.376 |  |  |
| 128 | LVKNKCVNF | B*08:01 | B8 | 533 | 541 | 0.34 |  |  |
| 129 | FIKQYGDCL | B*08:01 | B8 | 833 | 841 | 0.324 |  |  |
| 130 | DKVFRSSVL | B*08:01 | B8 | 40 | 48 | 0.306 |  |  |
| 131 | SAIGKIQDSL | B*08:01 | B8 | 929 | 938 | 0.28 |  | Vaccinated |
| 132 | VAKNLNESL | B*08:01 | B8 | 1189 | 1197 | 0.271 |  |  |
| 133 | DLPQGFSAL | B*08:01 | B8 | 215 | 223 | 0.267 |  | Naïve |
| 134 | NVVIKVCEF | B*08:01 | B8 | 125 | 133 | 0.214 |  |  |
| 135 | NSPRRARSV | B*08:01 | B8 | 679 | 687 | 0.199 |  | Vaccinated & N/I |
| 136 | DILSRLDKV | B*08:01 | B8 | 979 | 987 | 0.194 |  | Vaccinated |
| 137 | TTLDSKTQSL | B*08:01 | B8 | 108 | 117 | 0.192 |  |  |
| 138 | TLDSKTQSLL | B*08:01 | B8 | 109 | 118 | 0.179 |  |  |
| 139 | FKNLREFVF | B*08:01 | B8 | 186 | 194 | 0.177 |  |  |
| 140 | EAEVQIDRLI | B*49:01 | Other | 987 | 997 |  |  |  |
| 141 | RSFIEDLLF | B*58:01 | Other | 754 | 763 |  |  |  |
| 142 | VVNQNAQAL | A*26:01 | Other | 950 | 959 |  |  | Vaccinated |
| 143 | CVADYSVLY | A*26:01 | Other | 360 | 369 |  |  | Naïve |
| 144 | FEYVSQPFL | B*40:01 | Other | 167 | 176 |  |  |  |
| 145 | LEPLVDLPI | B*40:01 | Other | 222 | 231 |  |  |  |
| 146 | KRSFIEDLLFNKVTL | DPB1*02:01 | MHC II | 753 | 768 |  |  |  |
| 147 | NQKLIANQFNSAIGK | DRB1*13:02 | MHC II | 918 | 933 |  |  | Vaccinated |
| 148 | QKFNGLTVLPPLLTD | DRB1*01:01 | MHC II | 852 | 867 |  |  | Naïve |
| 149 | SSNFGAISSVLNDIL | DRB1*01:01DPA1*01:03 | MHC II | 966 | 981 |  |  | Naïve & N/I |
| 150 | CTFEYVSQPFLMDLE | DQB1*02:01DQB1*02:02DQB1*05:02DQB1*05:03DRB1*07:01DRB1*16:01 | MHC II | 165 | 180 |  |  |  |
| 151 | NIDGYFKIYSKHTPI | DRB1*07:01DRB1*15:01DRB1*16:01 | MHC II | 195 | 210 |  |  |  |
| 152 | LMDLEGKQGNFKNLR |  | MHC II | 175 | 192 |  |  | N/I |
| 153 | NFSQILPDPSKPSKR | DRB1*03:01 | MHC II | 800 | 815 |  |  |  |
| 154 | NLLLQYGSFCTQLNR | DQB1*05:03DRB1*04:04DRB1*15:01 | MHC II | 750 | 765 |  |  | N/I |
| 155 | CEFQFCNDPFLGVYY | DQB1*05:02DQB1*05:03 | MHC II | 130 | 145 |  |  | N/I |
| 156 | SSANNCTFEYVSQPF |  | MHC II | 160 | 175 |  |  | Naïve |
| 157 | NLVRDLPQGFSALEP | DRB1*03:01 | MHC II | 210 | 225 |  |  |  |
| 158 | RFASVYAWNRKRISN | DRB1*07:01DRB1*13:01 DRB1*14:01 | MHC II | 345 | 360 |  |  |  |
| 159 | AGFIKQYGDCLGDIA | DQB1*05:03 | MHC II | 830 | 845 |  |  |  |
| 160 | VSQPFLMDLEGKQGN | DRB1*03:01 | MHC II | 170 | 185 |  |  |  |
| 161 | EFVFKNIDGYFKIYS | DQB1*05:03DRB1*14:01 DRB1*15:01 | MHC II | 190 | 205 |  |  |  |
| 162 | FKIYSKHTPINLVRD | DRB1*07:01 DRB1*13:01 | MHC II | 200 | 215 |  |  |  |
| 163 | KHTPINLVRDLPQGF | DRB1*03:01 | MHC II | 204 | 219 |  |  | N/I |
| 164 | LPQGFSALEPLVDLP | DQB1*02:02DQB1*03:03DQB1*05:03 | MHC II | 215 | 230 |  |  | Naïve & N/I |
| 165 | TRFQTLLALHRSYLT | DQB1*05:03 DRB1*12:01 DRB1*14:01 | MHC II | 235 | 250 |  |  |  |
| 166 | IPFAMQMAYRFNGIG | DQB1*04:02DQB1*05:03 DRB1*12:01 DRB1*14:01 DRB1*15:01 | MHC II | 895 | 910 |  |  |  |
| 167 | KRISNCVADYSVLYN | DQB1*02:01 DQB1*02:02DRB1*03:01 | MHC II | 355 | 370 |  |  |  |
| 168 | LKPFERDISTEIYQA |  | MHC II | 460 | 475 |  |  |  |
| 169 | AENSVAYSNNSIAIP | DQB1*03:01 DRB1*15:01 | MHC II | 700 | 715 |  |  | Naïve |
| 170 | TNFTISVTTEILPVS | DQB1*02:02DQB1*06:03DRB1*07:01 DRB1*14:01 | MHC II | 715 | 730 |  |  | Naïve, & Vaccinated |

**Supplementary table 2. Donor characteristics and number of PBMCs stimulated per T cell peptide epitope**

The count of peripheral blood mononuclear cells (PBMCs) stimulated in each reaction was normalised across donor immune status of Naïve, Vaccinated or Naturally Infected, but varied between individual donors

| **Donor number** | **HLA type** | | | | **PBMCs / Stimulation (x10^5^)** | | |
| --- | --- | --- | --- | --- | --- | --- | --- |
|  | **HLA A** | | **HLA B** | | **Naïve** | **Vaccinated** | **Naturally infected** |
| 1 | A*24:02 | A*30:01 | B*07:02 | B*13:02 | 0.68 | 0.68 | 0.68 |
| 2 | A*02:01 | A*32:01 | B*51:01 | B*51:01 | 0.70 | 0.70 | 0.70 |
| 3 | A*01:01 | A*32:01 | B*08:01 | B*27:08 | 0.34 | 0.34 | 0.34 |
| 4 | A*24:02 | A*30:01 | B*07:02 | B*13:02 | 0.73 | 0.73 | 0.73 |
| 5 | A*11:01 | A*32:01 | B*51:06 | B*51:07 | 0.68 | 0.68 | 0.68 |
| 6 | A*02:01 | A*32:01 | B*51:01 | B*51:01 | 1.15 | 1.15 | 1.15 |
| 7 | A*01:01 | A*32:01 | B*08:01 | B*27:08 | 0.98 | NA | NA |
| 8 | A*01:01 | A*33:03 | B*13:02 | B*44:03 | 0.78 | 0.78 | NA |
| 9 | A*01:01 | A*30:04 | B*08:01 | B*27:05 | 0.97 | 0.97 | NA |
| 10 | A*02:01 | A*03:01 | B*44:02 | B*51:01 | NA | 1.02 | 1.02 |
| 11 | A*01:01 | A*11:01 | B*27:05 | B*35:03 | NA | 0.60 | 0.60 |

**Supplementary table 3. Immunoreactive peptide epitopes and previously reported immunogenicity**

Epitope immunoreactivity is reported as an increase in IFN-γ expression (ΔΔCt) after stimulation with a peptide epitope, compared to media-only stimulation. Data are presented for three groups: (i) naive, (ii) vaccinated, and (iii) naturally infected, and include the donor identification number (Donor ID) for instances in which ΔΔCt > 2 (shaded in grey). If an epitope has been previously demonstrated to stimulate human T cells in a particular group, a literature citation is provided. If no data are available or the epitope was found to not be immunoreactive, the entry is marked [Nil]. The table also shows the HLA allele that binds each epitope with highest affinity and the total number of references for that epitope on the Immune Epitope Database (IEDB), including any negative assay results. A summary of the immunoreactivity findings from the literature are also provided.

|  |  |  | **ΔΔCt**  **(Donor ID)**  **[Reference]^+^** | | |  |  |
| --- | --- | --- | --- | --- | --- | --- | --- |
| **ID** | **Peptide** | **HLA allele**  **(supertype)** | **Naïve** | **Vaccinated** | **Naturally infected** | **IEDB references*** | **Summary of literature** |
| 1 | HLMSFPQSA | A*02:01  (A2) | 2.93  (8)  [Nil] | -  -  [Nil] | -  -  ^2^ | 10 | In naturally infected individuals this epitope has been shown to be immunoreactive ^2^. It also exhibits high homology to the tumour-associated antigen LLWSFQTSA, which is immunoreactive in healthy individuals ^3^. Pre-existing epitope-specific T cells have been identified in naïve individuals ^4^. |
| 2 | YLQPRTFLL | A*02:01  (A2) | -  -  ^5^ | 4.50  (8)  ^6^ | -  -  ^2^ | 61 | This epitope is highly immunodominant ^7,8^ immunoreactive in the naïve ^5^ and elicits a robust response following vaccination ^6^, although not universally ^9^. It can also readily bind to several common HLA-C alleles ^10^. |
| 3 | VVFLHVTYV | A*02:03  (A2) | -  -  ^11^ | -  -  ^2^ | 2.94  (2)  ^2^ | 27 | This sub-dominant epitope can elicit responses in the naturally infected ^12-14^, and following vaccination ^1,11^. Pre-existing epitope-specific T cells have been identified in the naïve ^4^. |
| 5 | RLQSLQTYV | A*02:01  (A2) | 2.20  (1)  ^8^ | 3.24, 2.20  (3, 1)  ^15^ | -  -  ^2^ | 30 | This is a highly immunodominant epitope in the vaccinated ^6^ and naturally infected ^8^, and is occasionally immunoreactive in the naïve ^8^. A strong immunogenic response to this epitope has been linked to positive patient outcomes ^16^. |
| 6 | YQDVNCTEV | A*02:06  (A2) | -  -  [Nil] | 2.86  (3)  [Nil] | 2.06  (3)  ^2^ | 7 | This epitope is mutated in all commonly circulating SARS-COV-2 variants ^17^. Although pre-existing epitope-specific T cells have been identified in the naïve ^4^; this epitope was not immunoreactive following vaccination ^9^. |
| 7 | SIIAYTMSL | A*02:06  (A2) | -  -  ^5^ | 2.11  (1)  ^6^ | -  -  ^2^ | 28 | This epitope is immunoreactive in the vaccinated and naturally infected ^2,6^, as well as the naïve ^5^. Pre-existing epitope-specific T cells were identified in the naïve ^4^. |
| 8 | GLTVLPPLL | A*02:01  (A2) | 2.14  (8)  [Nil] | -  -  [Nil] | -  -  ^2^ | 6 | Has been found to be immunoreactive in convalescent COVID-19 patients ^2^. No specific-clonal prevalence has been identified in T cells from unexposed individuals ^4^. |
| 10 | VLNDILSRL | A*02:01  (A2) | 2.57  (8)  ^18^ | 2.78  (1)  ^1^ | -  -  ^19^ | 36 | This is a well-established sub-dominant epitope in the naturally infected ^19^. In 2009, this epitope was identified as immunoreactive in SARS-CoV 1 naïve donors ^20^ and is relatively highly conserved among other coronaviruses, including SARS-COV-2. When tested recently in the naïve, this epitope elicited a response ^18^ and a similar frequency of immunoreactive CD8^+^ T cells were found between naïve and convalescent donors ^21^. In contrast, others have also found no immunoreactive in the naïve ^13^ and no specific T cell clonal prevalence in the unexposed ^4^. This epitope is immunoreactive in various vaccination strategies ^1^, but not universally ^9^. |
| 13 | DISGINASV | A*68:02  (A2) | -  -  [Nil] | -  -  [Nil] | 2.14  (3)  [Nil] | 0 | Is within a CD4^+^ epitope LGDISGINASVVNIQ that has been found to be immunogenic within convalescent patients ^22^. But no immunogenicity data is available in the literature as a standalone CD8^+^ epitope. |
| 14 | KIADYNYKL | A*02:06  (A2) | 2.51  (1)  ^13^ | 2.11  (1)  ^23^ | -  -  ^24^ | 16 | Despite non-perfect homology, this epitope may provide cross-species reactivity to other endemic coronaviruses ^25^. Found to be immunoreactive in young convalescent patients but not old convalescent patients ^24^. Is immunoreactive in the naïve ^13^ and following vaccination ^23^, but not universally ^9^. |
| 16 | ALNTLVKQL | A*02:03  (A2) | 2.33  (8)  ^20^ | -  -  ^10^ | -  -  ^16^ | 16 | In 2009, this epitope was identified as immunoreactive in SARS-CoV 1 naïve donors ^20^. Sporadic responses identified it as subdominant following infection ^8^, while immunogenicity is linked to positive patient outcomes ^16^. Can bind to HLA-C alleles to elicit potent immunoreactivity following vaccination ^10^. |
| 26 | NSFTRGVYY | A*68:01  (A3/11) | 7.67  (8)  [Nil] | -  -  [Nil] | -  -  ^2^ | 4 | Sporadic responses identified this epitope as subdominant following infection ^8^. No specific information is available regarding immunoreactivity in the naïve. Multiple studies found this epitope was not immunoreactive following vaccination ^26,27^. |
| 29 | GVYFASTEK | A*68:01  (A3/11) | -  -  [Nil] | -  -  ^6^ | 2.05  (3)  ^2^ | 11 | Has been described as an immunodominant ^2^ and subdominant ^28^ epitope in convalescent patients ^14^. Has been found to elicit a response following vaccination ^6^. No data is available on immunoreactivity in the naïve. |
| 31 | GVYYPDKVFR | A*31:01  (A3/11) | 5.42, 3.08  (2, 8)  [Nil] | -  -  [Nil] | -  -  ^2^ | 3 | Immunoreactive in convalescent patients ^2^. The epitope ‘GVYYPDKVF’ is immunoreactive in various vaccination strategies ^1^. No data is available on full epitope immunoreactivity in the naïve or vaccinated. |
| 33 | GVYYHKNNK | A*03:01  (A3/11) | 17.06  (2)  [Nil] | -  -  ^23^ | -  -  ^2^ | 8 | Immunoreactive in convalescent patients ^2^, and following vaccination ^23^. No specific information is available regarding immunoreactivity in the naïve. |
| 35 | RASANLAATK | A*03:01  (A3/11) | -  -  [Nil] | 2.90  (1)  ^6^ | -  -  ^2^ | 4 | Immunoreactive in convalescent patients ^2^ and following vaccination ^6^. No specific information is available regarding immunoreactivity in the naïve. |
| 37 | HVTYVPAQEK | A*68:01  (A3/11) | -  -  [Nil] | 2.80  (1)  [Nil] | -  -  ^2^ | 1 | Immunoreactive in convalescent patients ^2^. No data is available on immunoreactivity in the naïve or vaccinated. |
| 41 | DGVYFASTEK | A*68:01  (A3/11) | 2.97  (8)  [Nil] | -  -  [Nil] | -  -  ^2^ | 1 | Immunoreactive in convalescent patients ^2^. No data is available on immunoreactivity in the naïve or vaccinated. |
| 42 | QTNSPRRAR | A*31:01  (A3/11) | -  -  [Nil] | -  -  [Nil] | 2.68  (3)  ^2^ | 3 | Immunoreactive in convalescent patients ^2^. Was found to not be immunoreactive following adenovirus vector vaccination ^11^. |
| 43 | SVYAWNRKR | A*31:01  (A3/11) | 4.62  (1)  [Nil] | 2.16  (1)  [Nil] | -  -  ^2^ | 7 | Immunoreactive in convalescent patients ^2^. Did not elicit a response following mRNA vaccination ^6^. No data is available on immunoreactivity in the naïve. |
| 48 | EYVSQPFLM | A*24:02  (A24) | 2.33  (8)  [Nil] | -  -  [Nil] | -  -  ^2^ | 7 | This epitope has been identified as immunoreactive in convalescent patients ^2^, in multiple studies ^29^. However, this epitope did not elicit a response following mRNA vaccination ^30^. No studies have tested the immunoreactivity of this epitope in naïve patients |
| 49 | VYSSANNCTF | A*24:02  (A24) | 3.29  (1)  [Nil] | 2.37  (1)  ^30^ | -  -  ^2^ | 7 | Immunoreactive in convalescent patients ^2^, and elicited a response following mRNA vaccination ^30^. Has been identified as an epitope with relatively high homology to other endemic coronaviruses ^31^. |
| 50 | IYQTSNFRV | A*24:02  (A24) | 2.60  (1)  [Nil] | -  -  [Nil] | -  -  ^2^ | 6 | Has been identified as immunoreactive in convalescent patients ^2^, in multiple studies ^29^. Did not elicit a response following mRNA vaccination ^30^. No data is available on immunoreactivity in the naïve. |
| 52 | HWFVTQRNF | A*24:02  (A24) | -  -  [Nil] | 6.21  (11)  [Nil] | 3.49  (6)  ^2^ | 6 | Has been identified as immunoreactive in convalescent patients ^2^, in multiple studies ^29^. Did not elicit a response following mRNA vaccination ^30^. No data is available on immunoreactivity in the naïve. |
| 54 | VFVSNGTHWF | A*24:02  (A24) | -  -  [Nil] | -  -  ^30^ | 2.91  (3)  ^2^ | 3 | Immunoreactive in convalescent patients ^2^, and following mRNA vaccination ^30^. No data is available on immunoreactivity in the naïve. Contains the epitope ‘FVSNGTHWF’ which is immunoreactive following vaccination ^1^. |
| 56 | RFDNPVLPF | A*24:02  (A24) | -  -  [Nil] | -  -  [Nil] | 2.17  (3)  ^2^ | 10 | Has been identified as immunoreactive in convalescent patients ^2^, in multiple studies ^14^, but did not elicit a response following mRNA vaccination ^30^. Has been identified an epitope with very low homology to other coronaviruses ^31^. |
| 57 | RVYSTGSNVF | A*24:02  (A24) | 2.60  (1)  ^13^ | 2.51  (1)  ^30^ | -  -  ^2^ | 4 | This epitope has been found to be immunoreactive in the naïve ^13^, following mRNA vaccination ^30^, and in convalescent patients ^2^. |
| 58 | YYHKNNKSW | A*23:01  (A24) | 3.01  (1)  [Nil] | -  -  [Nil] | -  -  ^2^ | 7 | A high-order nullomer (*i.e.,* a rarely occurring natural epitope) that binds strongly to eight common HLA and facilitates rapid HLA-Peptide processing (*i.e.,* TAP transport and protease action) ^32^. This epitope has been found to have immunoreactivity in convalescent patients ^2^, while others found no immunogenicity following vaccination ^30^. No data is available on immunoreactivity in the naïve. |
| 62 | TYVPAQEKNF | A*24:02  (A24) | 2.39  (3)  [Nil] | -  -  [Nil] | -  -  ^13^ | 4 | No specific study demonstrating immunoreactivity in the naïve, but the peptide has been identified as having a relatively higher homology to other endemic coronaviruses ^31^, and induces responses in the convalescent ^13^. All IEDB references report no immunogenicity following natural infection ^33^. |
| 65 | VYYPDKVF | A*24:02  (A24) | 2.37  (1)  [Nil] | -  -  [Nil] | -  -  ^2^ | 1 | Immunoreactive in convalescent patients ^2^. No data is available on immunoreactivity in the naïve or the vaccinated. |
| 68 | SFPQSAPHGVVF | A*24:02  (A24) | -  -  [Nil] | 4.29  (11)  [Nil] | -  -  ^2^ | 4 | Immunoreactive in convalescent patients ^2^, No data is available on immunoreactivity in the naïve or the vaccinated. Contains the epitope ‘QSAPHGVVF’ which is immunoreactive in various vaccination strategies ^1^. |
| 69 | YFPLQSYGF | A*29:02  (A24) | -  -  [Nil] | -  -  ^27^ | 4.13  (3)  ^34^ | 15 | Relatively well characterised A24 epitope ^35^ that has been identified as an immunodominant epitope in convalescent ^34^ and vaccinated individuals ^27^. |
| 71 | KPFERDIST | B*07:02  (B7) | 2.52  (1)  [Nil] | -  -  [Nil] | -  -  ^2^ | 0 | Immunoreactive in convalescent patients ^2^. No data is available on immunoreactivity in the naïve or the vaccinated. |
| 78 | QIPFAMQMAY | B*35:01  (B7) | -  -  [Nil] | -  -  [Nil] | 2.43  (1)  ^2^ | 1 | Immunoreactive in convalescent patients ^2^. No data is available on immunoreactivity in the naïve or the vaccinated. |
| 83 | LTDEMIAQY | B*35:01  (B7) | -  -  ^13^ | -  -  ^6^ | 2.70  (1)  ^14^ | 20 | Relatively well characterised epitope ^35^, which has been found to induce a response in convalescent patients ^14^, in multiple studies ^2,35^. Can elicit a response following mRNA vaccination ^6^, and in the naïve ^13^. Although, no epitope specific T cells were identified in the unexposed ^4^. |
| 85 | TSNQVAVLY | B*35:01  (B7) | -  -  [Nil] | 2.38  (3)  ^6^ | 2.45  (2)  ^2^ | 7 | Has been identified as immunoreactive in convalescent patients ^2^, and following vaccination ^6^. No data is available on immunoreactivity in the naïve; however, no epitope specific T cells were identified in the unexposed ^4^. |
| 86 | LPPLLTDEM | B*35:01  (B7) | -  -  [Nil] | 2.44  (2)  ^1^ | 2.41  (1)  ^2^ | 8 | Immunoreactive in convalescent patients ^2^, and the vaccinated ^1^, across various vaccination strategies ^1^. No data is available on immunoreactivity in the naïve. |
| 87 | IPTNFTISV | B*51:01  (B7) | 2.60  (1)  [Nil] | 2.51  (10)  [Nil] | -  -  ^2^ | 8 | Immunoreactive in convalescent patients ^2^. Despite carrying an alpha variant defining mutation it’s still likely to elicit a response ^36^. While pre-existing epitope-specific T cells were identified in the naïve ^4^, no response from naïve donors has been reported ^36^. |
| 91 | QPTESIVRF | B*51:01  (B7) | -  -  [Nil] | -  -  ^37^ | 5.09  (1)  ^2^ | 7 | Has been found to elicit a response following mRNA vaccination ^6^, in several studies ^37^, and is immunoreactive in convalescent patients ^2^. |
| 92 | SANNCTFEY | B*35:01  (B7) | -  -  [Nil] | 8.829  (10)  ^27^ | -  -  ^2^ | 4 | Immunoreactive in convalescent patients ^2^, and the vaccinated ^27^. Has the capacity to bind to many HLA alleles ^38^. No epitope specific T cells were identified in the unexposed ^4^. |
| 95 | DAVRDPQTL | B*51:01  (B7) | -  -  [Nil] | -  -  [Nil] | 2.85  (1)  ^2^ | 2 | Immunoreactivity was identified in convalescent patients ^2^. |
| 102 | QPRTFLLKY | B*35:01  (B7) | -  -  [Nil] | 3.97  (5)  [Nil] | 3.22, 2.45  (1, 3)  [Nil] | 2 | This epitope was not immunoreactive in convalescent patients ^34^, or in vaccinated mice ^39^. |
| 108 | VQPTESIVRF | B*53:01  (B7) | 2.83  (1)  [Nil] | -  -  [Nil] | 2.15  (1)  ^40^ | 1 | Identified as immunoreactive in convalescent patients following breakthrough infections ^40^. No data is available on immunoreactivity in the naïve or in the vaccinated. |
| 114 | IPFAMQMAY | B*35:01  (B7) | -  -  [Nil] | -  -  ^6^ | 2.01  (1)  ^2^ | 7 | Has been found to be immunoreactive in the convalescent ^2^, in multiple studies ^40,41^, and the vaccinated ^6^. No data is available on immunoreactivity in the naïve. |
| 119 | KIYSKHTPI | B*08:01  (B8) | -  -  [Nil] | -  -  [Nil] | 2.13  (3)  ^2^ | 3 | Immunoreactive in convalescent patients ^2^. No data is available on immunoreactivity in the naïve or the vaccinated. |
| 122 | DLLFNKVTL | B*08:01  (B8) | -  -  [Nil] | -  -  [Nil] | 3.77  (1)  [Nil] | 1 | This epitope was found to not be immunoreactive following natural infection in one study ^34^. No data is available on immunoreactivity in the naïve or the vaccinated. |
| 123 | LLALHRSYL | B*08:01  (B8) | -  -  [Nil] | -  -  ^42^ | 3.52  (1)  [Nil] | 3 | Epitope was identified as immunogenic following immunisation with a trailed peptide vaccine that generated strong T cell responses ^42^. Pre-existing epitope-specific T cells were identified in the naïve ^4^. No immunoreactivity data is available in the naïve or the naturally infected. |
| 131 | SAIGKIQDSL | B*08:01  (B8) | -  -  [Nil] | 3.70  (11)  [Nil] | -  -  [Nil] | 0 | No immunoreactivity data is available and there are no results in IEDB |
| 133 | DLPQGFSAL | B*08:01  (B8) | 2.80  (3)  [Nil] | -  -  [Nil] | -  -  [Nil] | 1 | Peptide aligns with sequences in the human proteome ^43^. Epitope was found to not be immunoreactive following inactivated virus vaccination ^9^. No immunoreactivity data has been recorded on IEDB. |
| 135 | NSPRRARSV | B*08:01  (B8) | -  -  [Nil] | 2.73  (1)  [Nil] | 2.41  (2)  [Nil] | 0 | In the BriSΔ variant of SARS-COV-2 this sequence was replaced with a single isoleucine (I), which significantly reduced transmissibility by abrogating S1/S2 cleavage ^44^. No immunoreactivity data has been recorded on IEDB. |
| 136 | DILSRLDKV | B*08:01  (B8) | -  -  [Nil] | 2.71  (1)  [Nil] | -  -  [Nil] | 0 | No immunoreactivity data is available and there are no results in IEDB. |
| 142 | VVNQNAQAL | A*26:01  (Other) | -  -  [Nil] | 7.56  (11)  ^1^ | -  -  [Nil] | 2 | Immunoreactive following various vaccination strategies ^1^. No immunoreactivity data is available in the naïve or the naturally infected. |
| 143 | CVADYSVLY | A*26:01  (Other) | 6.50  (9)  ^13^ | 3.51  (11)  ^27^ | -  -  ^2^ | 9 | Immunoreactive in the naïve ^13^, following vaccination ^27^, and in convalescent COVID-19 patients ^2^. |
| 145 | LEPLVDLPI | B*40:01  (Other) | 4.84  (4)  [Nil] | -  -  [Nil] | -  -  [Nil] | 1 | T cell binding assays were positive in convalescent COVID-19 patients ^45^. No data is available on epitope immunoreactivity. |
| 147 | NQKLIANQFNSAIGK | DRB1*13:02^#^  (Class II) | -  -  [Nil] | 7.56  (5)  [Nil] | -  -  [Nil] | 5 | No assays assessing immunogenicity are reported. However, this CD4^+^ epitope is readily presented on several MHC Class II alleles ^46^ and contains immunogenic CD8^+^ epitopes, such as the C*03:04 epitope ‘IANQFNSAI,’ which was found to be immunoreactive in various vaccination strategies ^1^. |
| 148 | QKFNGLTVLPPLLTD | DRB1*01:01^#^  (Class II) | 3.00  (1)  [Nil] | -  -  [Nil] | -  -  [Nil] | 11 | His epitope was found to be immunoreactive in HLA transgenic mice ^47^, however, no studies assessing human immunogenicity are reported. This peptide is readily presented on several MHC Class II alleles ^46^, and contains potentially immunoreactive epitopes such as A2 epitope ‘NGLTVLPPL’ ^9^. |
| 149 | SSNFGAISSVLNDIL | DRB1*01:01^#^  (Class II) | -  -  [Nil] | -  -  ^48^ | 3.51  (5)  ^48^ | 7 | Identified as readily presented on several MHC Class II alleles ^46^. Found to be immunoreactive in the vaccinated and subsequently convalescent ^48^. This response was preserved when infected with omicron despite the mutation ^48^. |
| 152 | LMDLEGKQGNFKNLR | (Class II) | -  -  [Nil] | -  -  ^1^ | 2.03  (4)  ^2^ | 8 | Have been identified as immunoreactive epitopes in vaccinated ^1^ and convalescent patients ^2^. |
| 154 | NLLLQYGSFCTQLNR | DQB1*05:03^#^  (Class II) | -  -  [Nil] | -  -  ^1^ | 2.59  (3)  ^49^ | 18 | Well characterized immunodominant Class II epitope, found to be immunoreactive following vaccination ^1,6^ and infection ^49,50^. |
| 155 | CEFQFCNDPFLGVYY | DQB1*05:02^#^  (Class II) | -  -  ^51^ | -  -  ^50^ | 2.00  (1)  ^50^ | 12 | Well characterized sub-dominant Class II epitope, found to be immunoreactive following vaccination and infection ^50^, and in the naïve ^51^. A2 epitope ‘CNDPFLGVYY’ was found to not be immunoreactive following inactivated virus vaccination ^9^; and A2 epitope ‘FCNDPFLGV’ was found to be immunoreactive in various vaccination strategies ^1^. |
| 156 | SSANNCTFEYVSQPF | (Class II) | 3.72  (1)  [Nil] | -  -  [Nil] | -  -  ^2^ | 17 | Found to be immunoreactive following infection ^2^ in multiple studies ^40,50^. Was found not immunoreactive following vaccination ^50^. Contains the A1 epitope ‘SSANNCTFEY’ shown to have immunoreactivity in the naïve ^13^. |
| 163 | KHTPINLVRDLPQGF | DRB1*03:01^#^  (Class II) | -  -  [Nil] | -  -  ^50^ | 5.08  (1)  ^6^ | 9 | Found to be immunoreactive following vaccination ^50^ and infection ^6,49^. Was found not immunoreactive in the naïve ^49^. |
| 164 | LPQGFSALEPLVDLP | DQB1*02:02^#^  (Class II) | 2.17  (1)  [Nil] | -  -  ^50^ | 7.47  (1)  ^2^ | 9 | Epitope shares pentapeptides with human proteins linked to oogenesis, placentation, and/or decidualization ^52^. B7:02 epitope ‘LPQGFSALEPL’ and B8:01 epitope ‘LPQGFSAL’ have both been shown to have immunoreactivity in the naïve ^13^. Immunoreactive following vaccination ^50^ and infection ^2^. |
| 169 | AENSVAYSNNSIAIP | DQB1*03:01^#^  (Class II) | 2.32  (7)  [Nil] | -  -  [Nil] | -  -  ^2^ | 15 | Identified as immunoreactive following infection ^2^. Found not to be immunoreactive following vaccination ^50^. |
| 170 | TNFTISVTTEILPVS | DQB1*02:02 ^#^  (Class II) | 2.82  (1)  [Nil] | 2.14  (9)  ^1^ | -  -  ^2^ | 10 | Well characterised immunodominant Class II epitope immunoreactive following vaccination ^50^ and infection ^1,2^. Contains the A2 epitope ‘FTISVTTEI’ which was immunoreactive in various immunisation strategies ^1^. |
|  | Positive Stimulations |  | 30 / 1500 | 26 / 1674 | 29 / 1360 |  |  |
|  | Positive Epitopes |  | 29 / 170 | 25 / 170 | 28 / 170 |  |  |

^+^ Selected reference demonstrating immunoreactivity within a specific donor group. ^*^ Collected 2024.07.23 ^#^ High affinity for multiple class II alleles

**Supplementary table 4. Categorical analysis of donor specific SARS-COV-2 S-protein epitope immunoreactivity following vaccination and natural infection**

The immunoreactivity from donors SARS-COV-2 S-protein naïve (Naïve), following COVID-19 vaccination (Vaccinated), and following infection with SARS-COV-2 (Naturally Infected) following S-protein peptide epitope stimulation. Immunoreactivity was quantified with high-throughput RT-qPCR (HTS-RT-qPCR) determining IFN-γ expression fold-change (ΔΔCt). Tested were the relative number of reactive stimulations (ΔΔCt > 2; Positive) to non-reactive stimulations (ΔΔCt < 2; Negative) across classes of immune status.

| **Donor** | **Immune status** | **Positive** | **Negative** | **Total** | ***P* value *vs.* naive** | ***P* value *vs.* vaccination** |
| --- | --- | --- | --- | --- | --- | --- |
| 1 | Naive | 15 | 155 | 170 | - | - |
|  | Vaccinated | 11 | 159 | 170 | 0.5412 | - |
|  | Naturally infected | 13 | 157 | 170 | 0.8440 | 0.8328 |
| 2 | Naive | 2 | 168 | 170 | - | - |
|  | Vaccinated | 1 | 169 | 170 | >0.9999 | - |
|  | Naturally infected | 3 | 167 | 170 | >0.9999 | 0.6228 |
| 3 | Naive | 2 | 168 | 170 | - | - |
|  | Vaccinated | 3 | 167 | 170 | >0.9999 | - |
|  | Naturally infected | 10 | 160 | 170 | 0.0353 | 0.0861 |
| 4 | Naive | 1 | 169 | 170 | - | - |
|  | Vaccinated | 0 | 170 | 170 | >0.9999 | - |
|  | Naturally infected | 1 | 169 | 170 | >0.9999 | >0.9999 |
| 5 | Naive | 0 | 170 | 170 | - | - |
|  | Vaccinated | 2 | 168 | 170 | 0.4985 | - |
|  | Naturally infected | 1 | 169 | 170 | >0.9999 | >0.9999 |
| 6 | Naive | 0 | 170 | 170 | - | - |
|  | Vaccinated | 0 | 170 | 170 | >0.9999 | - |
|  | Naturally infected | 1 | 169 | 170 | >0.9999 | >0.9999 |
| 7 | Naive | 1 | 169 | 170 | - | - |
|  | Vaccinated | - | - | - | - | - |
|  | Naturally infected | - | - | - | - | - |
| 8 | Naive | 8 | 162 | 170 | - | - |
|  | Vaccinated | 1 | 169 | 170 | 0.0366 | - |
|  | Naturally infected | - | - | - | - | - |
| 9 | Naive | 1 | 169 | 170 | - | - |
|  | Vaccinated | 1 | 169 | 170 | >0.9999 | - |
|  | Naturally infected | - | - | - | - | - |
| 10 | Naive | - | - | - | - | - |
|  | Vaccinated | 2 | 168 | 170 | - | - |
|  | Naturally infected | 0 | 170 | 170 | - | 0.4985 |
| 11 | Naive | - | - | - | - | - |
|  | Vaccinated | 5 | 165 | 170 | - | - |
|  | Naturally infected | 0 | 170 | 170 | - | 0.0607 |

**Supplementary table 5. Categorical analysis of HLA restriction grouped SARS-COV-2 S‑protein epitope immunoreactivity following vaccination and natural infection**

The combined (n=12) immunoreactivity from donors SARS-COV-2 S-protein naïve (Naïve), following COVID-19 vaccination (Vaccinated), and following infection with SARS-COV-2 (Naturally infected) following S‑protein peptide epitope stimulation. Immunoreactivity was quantified with high-throughput RT‑qPCR (HTS-RT-qPCR) determining IFN-γ expression fold-change (ΔΔCt). Tested were the relative number of reactive stimulations (ΔΔCt > 2; Positive) to non-reactive stimulations (ΔΔCt < 2; Negative) across classes of immune status, considering the predicted peptide HLA restriction (Peptide HLA).

| **Peptide HLA** | **Status** | **Positive** | **Negative** | ***P* value* *vs.* naive** | ***P* value* *vs.* post-vaccination** |
| --- | --- | --- | --- | --- | --- |
| A2 | Naive | 6 | 201 | - | - |
|  | Vaccinated | 7 | 224 | >0.9999 | - |
|  | Naturally infected | 3 | 182 | 0.5091 | 0.5226 |
| A3/11 | Naive | 6 | 201 | - | - |
|  | Vaccinated | 3 | 227 | 0.3185 | - |
|  | Naturally infected | 2 | 182 | 0.2909 | >0.9999 |
| A24 | Naive | 7 | 200 | - | - |
|  | Vaccinated | 4 | 226 | 0.3631 | - |
|  | Naturally infected | 4 | 180 | 0.5510 | >0.9999 |
| B7 | Naive | 3 | 419 | - | - |
|  | Vaccinated | 5 | 464 | 0.7284 | - |
|  | Naturally infected | 10 | 365 | 0.0462 | 0.1139 |
| B8 | Naive | 1 | 206 | - | - |
|  | Vaccinated | 3 | 227 | 0.6254 | - |
|  | Naturally infected | 4 | 180 | 0.1920 | 0.7048 |
| Other | Naive | 2 | 52 | - | - |
|  | Vaccinated | 2 | 58 | >0.9999 | - |
|  | Naturally infected | 0 | 48 | 0.4968 | 0.5016 |
| Class II | Naive | 5 | 220 | - | - |
|  | Vaccinated | 2 | 248 | 0.2640 | - |
|  | Naturally infected | 6 | 194 | 0.1469 | 0.7622 |
| HLA - A | Naive | 21 | 654 |  |  |
|  | Vaccinated | 16 | 735 | 0.2491 |  |
|  | Naturally infected | 9 | 592 | 0.0650 | 0.4240 |
| HLA - B | Naive | 4 | 625 |  |  |
|  | Vaccinated | 8 | 691 | 0.3936 |  |
|  | Naturally infected | 14 | 545 | 0.0149 | 0.0675 |
| HLA  A *vs.* B | Naïve *vs.* naïve | | | *P =* 0.0010 |  |
|  | Vaccinated *vs.* vaccinated | | | *P =* 0.1545 |  |
|  | Naturally infected *vs*. naturally infected | | | *P =* 0.2921 |  |

**Supplementary table 6. Categorical analysis of homology of immunoreactive SARS-COV-2 S-protein peptide epitopes following vaccination and natural infection**

The homology of immunoreactive epitopes (ΔΔCt > 2) were tested relative to the number of epitopes with complete homology (100% Homology) and those containing at least one variable amino acid, in at least one SARS-COV-2 variant (<100% Homology) across combined (n=12) donors of various immune statuses. Epitopes that were positive across multiple donors of the same immune status were counted once.

| **Immune status** | **Homology< 100%** | **100% = Homology** | **Total** | **Homology < 100% (%)** | ***P* value *vs.* naive** | ***P* value *vs.* vaccination** | ***P* value *vs.* naturally infected** |
| --- | --- | --- | --- | --- | --- | --- | --- |
| Total peptides | 38 | 132 | 170 | 22.3% | 0.6336 | 0.3154 | 0.1449 |
| Naïve | 8 | 21 | 29 | 27.6% |  |  |  |
| Vaccinated | 8 | 17 | 25 | 32.0% | 0.7718 |  |  |
| Naturally infected | 10 | 17 | 27 | 37.0% | 0.5695 | 0.7756 |  |

**Supplementary table 7. Categorical analysis of immunoreactive epitope localization within SARS-COV-2 S-protein domains following vaccination and natural infection**

The location of immunoreactive epitopes (ΔΔCt > 2) were tested relative to the number of epitopes inside and outside of domains across combined (n=12) donors of various immune statuses. Tested were Sub-Domain 1 (1‑681 aa), and Sub-Domain 2 (686-1273 aa), and the, Receptor Binding Motif (438-508 aa), the RAAR Motif (682-685aa), the S1/S2 Cleavage Site (672-709 aa), the Fusion Peptide (788-806 aa), the Internal Fusion Peptide (816-833 aa), Heptad Repeat 1 (981-983 aa), Heptad Repeat 2 (1162-1203 aa), and peptide epitopes that were in do discreetly defined regions (No Region).

| **Domain** | **Immune status** | **Epitopes in domain** | **Epitopes outside domain** | **Total** | **In domain (%)** | ***P* value *vs.* naive** | ***P* value *vs.* vaccination** |
| --- | --- | --- | --- | --- | --- | --- | --- |
| Sub Domain 1 | Naïve | 20 | 10 | 30 | 66.6% | - |  |
|  | Vaccinated | 11 | 15 | 26 | 42.3% | 0.1056 | - |
|  | Naturally infected | 18 | 11 | 29 | 62.1% | 0.1811 | 0.7892 |
| Sub Domain 2 | Naïve | 10 | 20 | 30 | 33.3% | - |  |
|  | Vaccinated | 15 | 11 | 26 | 57.7% | 0.1056 | - |
|  | Naturally infected | 11 | 18 | 29 | 37.9% | 0.1811 | 0.7892 |
| Receptor Binding Motif | Naïve | 1 | 29 | 30 | 3.3% | - |  |
|  | Vaccinated | 0 | 26 | 26 | 0.0% | >0.9999 | - |
|  | Naturally infected | 1 | 28 | 29 | 3.4% | >0.9999 | >0.9999 |
| RAAR Motif | Naïve | 0 | 30 | 30 | 0.0% | - |  |
|  | Vaccinated | 0 | 26 | 26 | 0.0% | >0.9999 | - |
|  | Naturally infected | 0 | 29 | 29 | 0.0% | >0.9999 | >0.9999 |
| S1/S2 Cleavage Site | Naïve | 1 | 29 | 30 | 3.3% | - |  |
|  | Vaccinated | 2 | 24 | 26 | 7.7% | 0.5920 | - |
|  | Naturally infected | 2 | 27 | 29 | 6.9% | 0.6120 | >0.9999 |
| Fusion Peptide | Naïve | 0 | 30 | 30 | 0.0% | - |  |
|  | Vaccinated | 0 | 26 | 26 | 0.0% | >0.9999 | - |
|  | Naturally infected | 0 | 29 | 29 | 0.0% | >0.9999 | >0.9999 |
| Internal Fusion Peptide | Naïve | 0 | 30 | 30 | 0.0% | - |  |
|  | Vaccinated | 0 | 26 | 26 | 0.0% | >0.9999 | - |
|  | Naturally infected | 1 | 28 | 29 | 3.5% | 0.4915 | >0.9999 |
| Heptad Repeat 1 | Naïve | 2 | 28 | 30 | 0.06% | - |  |
|  | Vaccinated | 4 | 22 | 26 | 15.4% | 0.4006 | - |
|  | Naturally infected | 1 | 28 | 29 | 3.5% | >0.9999 | 0.1777 |
| Heptad Repeat 2 | Naïve | 0 | 30 | 30 | 0.0% | - |  |
|  | Vaccinated | 0 | 26 | 26 | 0.0% | >0.9999 | - |
|  | Naturally infected | 1 | 28 | 29 | 3.5% | 0.4915 | >0.9999 |
| No Region | Naïve | 8 | 22 | 30 | 26.7% | - |  |
|  | Vaccinated | 13 | 13 | 26 | 50.0% | 0.0990 | - |
|  | Naturally infected | 10 | 19 | 29 | 34.5% | 0.5796 | 0.2832 |

**References within this SUPPORTING INFORMATION FILE**

1. Tarke A, Coelho CH, Zhang Z*, et al.* SARS-COV-2 vaccination induces immunological T cell memory able to cross-recognize variants from Alpha to Omicron. *Cell* 2022*;***185**: 847-859.

2. Tarke A, Sidney J, Kidd CK*, et al.* Comprehensive analysis of T cell immunodominance and immunoprevalence of SARS-COV-2 epitopes in COVID-19 cases. *Cell Rep Med* 2021; **2**: 100204.

3. Ragone C, Mauriello A, Cavalluzzo B*, et al.* Molecular mimicry of SARS-COV-2 antigens as a possible natural anti-cancer preventive immunization. *Front Immunol* 2024; **15**: 1398002.

4. Francis JM, Leistritz-Edwards D, Dunn A*, et al.* Allelic variation in class I HLA determines CD8^+^ T cell repertoire shape and cross-reactive memory responses to SARS-COV-2. *Sci Immunol* 2022; **7**: eabk3070.

5. Quiros-Fernandez I, Poorebrahim M, Fakhr E, Cid-Arregui A. Immunogenic T cell epitopes of SARS-COV-2 are recognized by circulating memory and naive CD8 T cells of unexposed individuals. *eBioMedicine* 2021; **72**: 103610.

6. Lang-Meli J, Luxenburger H, Wild K*, et al.* SARS-COV-2-specific T-cell epitope repertoire in convalescent and mRNA-vaccinated individuals. *Nat Microbiol* 2022: **7**: 675-679.

7. Szeto C, Nguyen AT, Lobos CA*, et al.* Molecular Basis of a Dominant SARS-COV-2 Spike-Derived Epitope Presented by HLA-A*02:01 Recognised by a Public TCR. *Cells* 2021; **10**: 10102646

8. Shomuradova AS, Vagida MS, Sheetikov SA*, et al.* SARS-COV-2 Epitopes Are Recognized by a Public and Diverse Repertoire of Human T Cell Receptors. *Immunity* 2020; **53**: 1245-1257

9. Xiao C, Ren Z, Zhang B*, et al.* Insufficient epitope-specific T cell clones are responsible for impaired cellular immunity to inactivated SARS-COV-2 vaccine in older adults. *Nat Aging* 2023; **3**: 418-435.

10. Wang Y, Yang L, Tang K*, et al.* Ad5-nCoV Vaccination Could Induce HLA-E Restricted CD8^+^ T Cell Responses Specific for Epitopes on *Severe Acute Respiratory Syndrome Coronavirus 2* Spike Protein. *Viruses* 2023; **16**: 16010052.

11. Titov A, Shaykhutdinova R, Shcherbakova OV*, et al.* Immunogenic epitope panel for accurate detection of non-cross-reactive T cell response to SARS-COV-2. *JCI Insight* 2022; **7**: 157699

12. Qiu C, Xiao C, Wang Z*, et al.* CD8^+^ T-Cell Epitope Variations Suggest a Potential Antigen HLA-A2 Binding Deficiency for Spike Protein of SARS-COV-2. *Front Immunol* 2021; **12**: 764949.

13. Saini SK, Hersby DS, Tamhane T*, et al.* SARS-COV-2 genome-wide T cell epitope mapping reveals immunodominance and substantial CD8^+^ T cell activation in COVID-19 patients. *Sci Immunol* 2021; **6**: abf7550.

14. Kared H, Redd AD, Bloch EM*, et al.* SARS-COV-2-specific CD8^+^ T cell responses in convalescent COVID-19 individuals. *J Clin Invest* 2021; **131**: 45476.

15. Sheetikov SA, Khmelevskaya AA, Zornikova KV*, et al.* Clonal structure and the specificity of vaccine-induced T cell response to SARS-COV-2 Spike protein. *Front Immunol* 2024; **15**: 1369436.

16. Coulon PG, Prakash S, Dhanushkodi NR*, et al.* High frequencies of alpha common cold coronavirus/SARS-COV-2 cross-reactive functional CD4^+^ and CD8^+^ memory T cells are associated with protection from symptomatic and fatal SARS-COV-2 infections in unvaccinated COVID-19 patients. *Front Immunol* 2024; **15**: 1343716.

17. Hamelin DJ, Fournelle D, Grenier JC*, et al.* The mutational landscape of SARS-COV-2 variants diversifies T cell targets in an HLA-supertype-dependent manner. *Cell Syst* 2022; **13**: 143-157

18. Prakash S, Srivastava R, Coulon PG*, et al.* Genome-Wide B Cell, CD4^+^, and CD8^+^ T Cell Epitopes That Are Highly Conserved between Human and Animal Coronaviruses, Identified from SARS-COV-2 as Targets for Preemptive Pan-Coronavirus Vaccines. *J Immunol* 2021; **206**: 2566-2582.

19. Habel JR, Nguyen THO, van de Sandt CE*, et al.* Suboptimal SARS-COV-2-specific CD8^+^ T cell response associated with the prominent HLA-A*02:01 phenotype. *Proc Natl Acad Sci USA* 2020; **117**: 24384-24391.

20. Lv Y, Ruan Z, Wang L, Ni B, Wu Y. Identification of a novel conserved HLA-A*0201-restricted epitope from the spike protein of SARS-CoV. *BMC Immunol* 2009; **10**: 61.

21. Maino A, Amen A, Plumas J*, et al.* Development of a New Off-the-Shelf Plasmacytoid Dendritic Cell–Based Approach for the Expansion and Characterization of SARS-COV-2–Specific T Cells. *J Immunol* 2024; **212**: 825-833.

22. Mateus J, Grifoni A, Tarke A*, et al.* Selective and cross-reactive SARS-COV-2 T cell epitopes in unexposed humans. *Science* 2020; **370**: 89-94.

23. Zhang H, Deng S, Ren L*, et al.* Profiling CD8^+^ T cell epitopes of COVID-19 convalescents reveals reduced cellular immune responses to SARS-COV-2 variants. *Cell Rep* 2021; **36**: 109708.

24. Proietto D, Dallan B, Gallerani E*, et al.* Ageing Curtails the Diversity and Functionality of Nascent CD8^+^ T Cell Responses against SARS-COV-2. *Vaccines* 2023; **11**: 11010154.

25. Antonio EC, Meireles MR, Bragatte MAS, Vieira GF. Viral immunogenic footprints conferring T cell cross-protection to SARS-COV-2 and its variants. *Front Immunol* 2022; **13**: 931372.

26. Lie-Andersen O, Hubbe ML, Subramaniam K*, et al.* Impact of peptide:HLA complex stability for the identification of SARS-COV-2-specific CD8^+^ T cells. *Front Immunol* 2023; **14**: 1151659.

27. Swaminathan S, Lineburg KE, Panikkar A*, et al.* Ablation of CD8^+^ T cell recognition of an immunodominant epitope in SARS-COV-2 Omicron variants BA.1, BA.2 and BA.3. *Nat Commun* 2022; **13**: 6387.

28. Redd AD, Nardin A, Kared H*, et al.* Minimal Crossover between Mutations Associated with Omicron Variant of SARS-COV-2 and CD8^+^ T-Cell Epitopes Identified in COVID-19 Convalescent Individuals. *mBio* 2022; **13**: e03617-21.

29. Hu C, Shen M, Han X*, et al.* Identification of cross-reactive CD8^+^ T cell receptors with high functional avidity to a SARS-COV-2 immunodominant epitope and its natural mutant variants. *Genes Dis* 2022; **9**: 216-229.

30. Kuse N, Zhang Y, Chikata T*, et al.* Long-term memory CD8^+^ T cells specific for SARS-COV-2 in individuals who received the BNT162b2 mRNA vaccine. *Nat Commun* 2022; **13**: 5251.

31. Pushpakumara PD, Madhusanka D, Dhanasekara S*, et al.* Identification of Novel Candidate CD8^+^ T Cell Epitopes of the SARS-CoV *2* with Homology to Other Seasonal Coronaviruses. *Viruses* 2021; **13**: 972

32. Santoni D, Vergni D. In the search of potential epitopes for Wuhan seafood market pneumonia virus using high order nullomers. *J Immunol Methods* 2020; **481**: 112787.

33. Shimizu K, Iyoda T, Sanpei A*, et al.* Identification of TCR repertoires in functionally competent cytotoxic T cells cross-reactive to SARS-COV-2. *Commun Biol* 2021; **4**: 1365.

34. Gfeller D, Schmidt J, Croce G*, et al.* Improved predictions of antigen presentation and TCR recognition with MixMHCpred2.2 and PRIME2.0 reveal potent SARS-COV-2 CD8^+^ T-cell epitopes. *Cell Syst* 2023; **14**: 72-83.

35. Jin X, Liu X, Shen C. A systemic review of T-cell epitopes defined from the proteome of SARS-COV-2. *Virus Res* 2023; **324**: 199024.

36. Gangaev A, Ketelaars SLC, Isaeva OI*, et al.* Identification and characterization of a SARS-COV-2 specific CD8^+^ T cell response with immunodominant features. *Nat Commun* 2021; **12**: 2593.

37. Oberhardt V, Luxenburger H, Kemming J*, et al.* Rapid and stable mobilization of CD8^+^ T cells by SARS-COV-2 mRNA vaccine. *Nature* 2021; **597**: 268-273.

38. Gustiananda M, Julietta V, Hermawan A*, et al.* Immunoinformatics Identification of the Conserved and Cross-Reactive T-Cell Epitopes of SARS-COV-2 with Human Common Cold Coronaviruses, SARS-CoV-2, MERS-CoV and Live Attenuated Vaccines Presented by HLA Alleles of Indonesian Population. *Viruses* 2022; **14**: 2328.

39. Wang Y, Wang B, Zhao Z*, et al.* Effects of SARS-COV-2 Omicron BA.1 Spike Mutations on T-Cell Epitopes in Mice. *Viruses* 2023; **15**: 15030763.

40. Tarke A, Ramezani-Rad P, Alves Pereira Neto T*, et al.* SARS-COV-2 breakthrough infections enhance T cell response magnitude, breadth, and epitope repertoire. *Cell Rep Med* 2024; **5**: 101583.

41. Lineburg KE, Crooks P, Raju J*, et al.* Breakthrough SARS-COV-2 infection induces broad anti-viral T cell immunity. *iScience* 2023; **26**: 108474.

42. Heitmann JS, Bilich T, Tandler C*, et al.* A COVID-19 peptide vaccine for the induction of SARS-COV-2 T cell immunity. *Nature* 2022; **601**: 617-622.

43. Felipe Cuspoca A, Isaac Estrada P, Velez-van-Meerbeke A. Molecular Mimicry of SARS-COV-2 Spike Protein in the Nervous System: A Bioinformatics Approach. *Comput Struct Biotechnol J* 2022; **20**: 6041-6054.

44. Bamford CGG, Broadbent L, Aranday-Cortes E*, et al.* Comparison of SARS-COV-2 Evolution in Paediatric Primary Airway Epithelial Cell Cultures Compared with Vero-Derived Cell Lines. *Viruses* 2022; **14:** 325.

45. Snyder TM, Gittelman RM, Klinger M*, et al.* Magnitude and dynamics of the T-cell response to SARS-COV-2 infection at both individual and population levels. *Front Immunol* 2025; **7**:15:1488860.

46. Obermair FJ, Renoux F, Heer S*, et al.* High-resolution profiling of MHC II peptide presentation capacity reveals SARS-COV-2 CD4 T cell targets and mechanisms of immune escape. *Sci Adv* 2022; **8**: eabl5394.

47. Dos Santos Alves RP, Timis J, Miller R*, et al.* Human coronavirus OC43-elicited CD4^+^ T cells protect against SARS-COV-2 in HLA transgenic mice. *Nat Commun* 2024; **15**: 787.

48. Emmelot ME, Vos M, Boer MC*, et al.* Omicron BA.1 Mutations in SARS-COV-2 Spike Lead to Reduced T-Cell Response in Vaccinated and Convalescent Individuals. *Viruses* 2022; **14**: 1570.

49. Peng Y, Mentzer AJ, Liu G*, et al.* Broad and strong memory CD4^+^ and CD8^+^ T cells induced by SARS-COV-2 in UK convalescent individuals following COVID-19. *Nat Commun* 2020; **21**: 1336-1345.

50. Karsten H, Cords L, Westphal T*, et al.* High-resolution analysis of individual spike peptide-specific CD4^+^ T-cell responses in vaccine recipients and COVID-19 patients. *Clin Transl Immunol* 2022; **11**: e1410.

51. Zhang Y, Yang Z, Tang M*, et al.* Three Specific Potential Epitopes That Could Be Recognized by T Cells of Convalescent COVID-19 Patients Were Identified From Spike Protein. *Front Immunol* 2022; **13**: 752622.

52. Dotan A, Kanduc D, Muller S, Makatsariya A, Shoenfeld Y. Molecular mimicry between SARS-COV-2 and the female reproductive system. *Am J Reprod Immunol* 2021; **86**: e13494.
